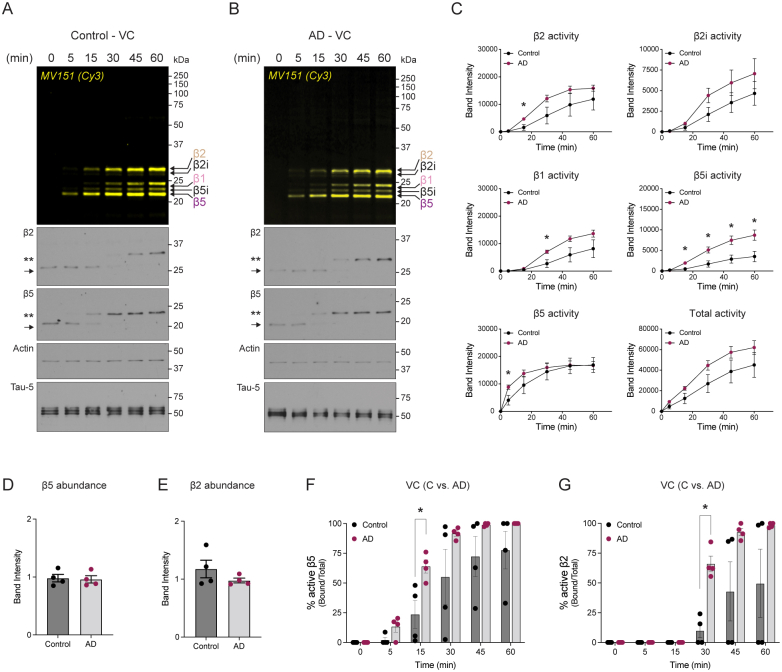# Orthogonal approaches required to measure proteasome composition and activity in mammalian brain tissue

**DOI:** 10.1016/j.jbc.2023.104811

**Published:** 2023-05-11

**Authors:** Fulya Türker, Rahul A. Bharadwaj, Joel E. Kleinman, Daniel R. Weinberger, Thomas M. Hyde, Cory J. White, Dionna W. Williams, Seth S. Margolis

**Affiliations:** 1 Department of Biological Chemistry, The Johns Hopkins University School of Medicine, Baltimore, Maryland, USA; 2 The Lieber Institute for Brain Development, Baltimore, Maryland, USA; 3 Department of Psychiatry and Behavioral Sciences, The Johns Hopkins University School of Medicine, Baltimore, Maryland, USA; 4 McKusick-Nathans Institute of Genetic Medicine, The Johns Hopkins University School of Medicine, Baltimore, Maryland, USA; 5 Department of Neurology, The Johns Hopkins University School of Medicine, Baltimore, Maryland, USA; 6 Department of Molecular and Comparative Pathobiology, The Johns Hopkins University School of Medicine, Baltimore, Maryland, USA; 7 Department of Pharmacology and Molecular Sciences, The Johns Hopkins University School of Medicine, Baltimore, Maryland, USA; 8 Division of Clinical Pharmacology, Department of Medicine, Johns Hopkins School of Medicine, Baltimore, Maryland, USA; 9 Department of Molecular Microbiology & Immunology, Johns Hopkins School of Public Health, Baltimore, Maryland, USA; 10 Solomon H. Snyder Department of Neuroscience, The Johns Hopkins University School of Medicine, Baltimore, Maryland, USA

**Keywords:** proteasome, protein degradation, activity-based probe, MV151, Suc-LLVY-AMC, mammalian central nervous system, neuron, neurodegeneration, Alzheimer's disease

## Abstract

Proteasomes are large macromolecular complexes with multiple distinct catalytic activities that are each vital to human brain health and disease. Despite their importance, standardized approaches to investigate proteasomes have not been universally adapted. Here, we describe pitfalls and define straightforward orthogonal biochemical approaches essential to measure and understand changes in proteasome composition and activity in the mammalian central nervous system. Through our experimentation in the mammalian brain, we determined an abundance of catalytically active proteasomes exist with and without a 19S cap(s), the regulatory particle essential for ubiquitin-dependent degradation. Moreover, we learned that in-cell measurements using activity-based probes (ABPs) are more sensitive in determining the available activity of the 20S proteasome without the 19S cap and in measuring individual catalytic subunit activities of each β subunit within all neuronal proteasomes. Subsequently, applying these tools to human brain samples, we were surprised to find that post-mortem tissue retained little to no 19S-capped proteasome, regardless of age, sex, or disease state. In comparing brain tissues (parahippocampal gyrus) from patients with Alzheimer’s disease (AD) and unaffected individuals, the available 20S proteasome activity was significantly elevated in severe cases of AD, an observation not previously noted. Taken together, our study establishes standardized approaches for the comprehensive investigation of proteasomes in mammalian brain tissue, and we reveal new insight into brain proteasome biology.

The maintenance of cellular health depends on the regulated clearance of proteins through proteasomes. Proteasomes accomplish this task by degrading the bulk of ubiquitin-tagged proteins (1) that are folded, unstructured, or aggregated (2, 3) as well as non-ubiquitylated unfolded proteins (4, 5). Interference of proteasomes perturbs the clearance of these end products of proteotoxic stress. This alters intracellular processes required for proper cellular function and ultimately contributes to age-related decline in cell and organismal health (1, 4, 6, 7). In the mammalian nervous system, for example, total inhibition of all proteasome complexes disrupts neuronal cell migration (8, 9), synaptic remodeling (10, 11), physiology (12, 13, 14, 15, 16, 17, 18, 19, 20), and cell survival (21). Moreover, human studies have shown an association between defects in proteasome function and brain disease (1, 6), including neurodegeneration (22, 23, 24, 25, 26, 27, 28, 29). Consistent with this, Alzheimer’s disease (AD) causing β–amyloid protein (30) (Aβ) has been shown to inhibit protein degradation pathways (26, 28, 29, 31, 32, 33) and promote defects in protein clearance leading to increased protein aggregation (33, 34, 35, 36, 37, 38). Collectively, these studies conclude that there is a link between proteasome function in the nervous system and its potential contribution to the pattern and severity of cognitive decline observed in AD and possibly other neurodegenerative disorders (1, 21, 39, 40). As a result of the conclusions made from these studies, an emerging effort has been to find new ways to activate proteasomes with the hope of slowing down or reversing some of the demise of brain disease observed across various neurodegenerative disorders (41, 42, 43). Despite a considerable effort to understand proteasome biology, deep insight into the composition and activity of proteasomes in the mammalian nervous system and human brain diseases has been limited to only a few measurements and at times single assays. The proteasome, however, is more complex than generally appreciated and, we believed, required a closer look at the mammalian nervous system.

Proteasomes are composed of 28 subunits (α7β7β7α7) with an architecture of four heptameric rings (Fig. 1*A*) (5, 44, 45, 46, 47). Within this 719-kDa core particle exists three catalytically active proteasome β subunits (β1, β2, β5), which have distinct activities and work together to degrade intracellular proteins (48). This core particle is referred to as the 20S proteasome and can be associated with a multisubunit complex that makes up the 19S regulatory cap to form a 26S (one cap, ∼1.5 MDa) or 30S (two caps, ∼2.5 MDa) proteasome (5, 46, 49, 50, 51). Proteasomes can also associate with a variety of regulator and activator complexes that can modulate proteasome activity and promote the opening of the 20S proteasome (52, 53, 54, 55). While the natural behavior of the 19S-capped proteasomes is to mediate ATP-dependent unfolding and degradation of ubiquitylated proteins, the 20S proteasome without the 19S cap does not require ubiquitin or ATP and is thought to be primarily tasked with degrading already unfolded proteins (Note: Unless stated otherwise, when discussing 20S proteasome, we refer to a 20S proteasome without a 19S cap) (4, 7, 56). In the nervous system, 19S-capped proteasomes are the main focus despite many studies relying largely on pan-proteasome inhibitors and single assay measurements, which do not distinguish between the various proteasome complexes (8, 9, 10, 11, 12, 13, 14, 15, 16, 17, 18, 19, 20, 57). In fact, prior evidence indicates that 20S proteasome is the predominant form of the proteasome in cells, comprising ∼64% of the entire cellular proteasome pool (56, 58). Moreover, similar studies have also shown that roughly 40% to 50% of proteasomes are not 19S-capped in the mouse nervous system (59). While these several studies were done in cell culture, our understanding of proteasome composition and activity in mammalian brain tissue remains largely unexplored. Given that proteasome composition can alter the state of intracellular degradation of protein substrates which gives rise to an ever-changing pool of peptide products (56, 59, 60), we considered that a deeper understanding of the relative expression and activity of various proteasome complexes in the nervous system would provide key insight into changes to proteostasis. Understanding more about these changes in the proteostasis network will contribute to interventions to improve neurological decline due to aberrant proteasome function. Thus, to study proteasomes in the mammalian brain, we explored the utility of multiple orthogonal biochemical approaches that have been used for decades across multiple systems.

**Figure 1:**
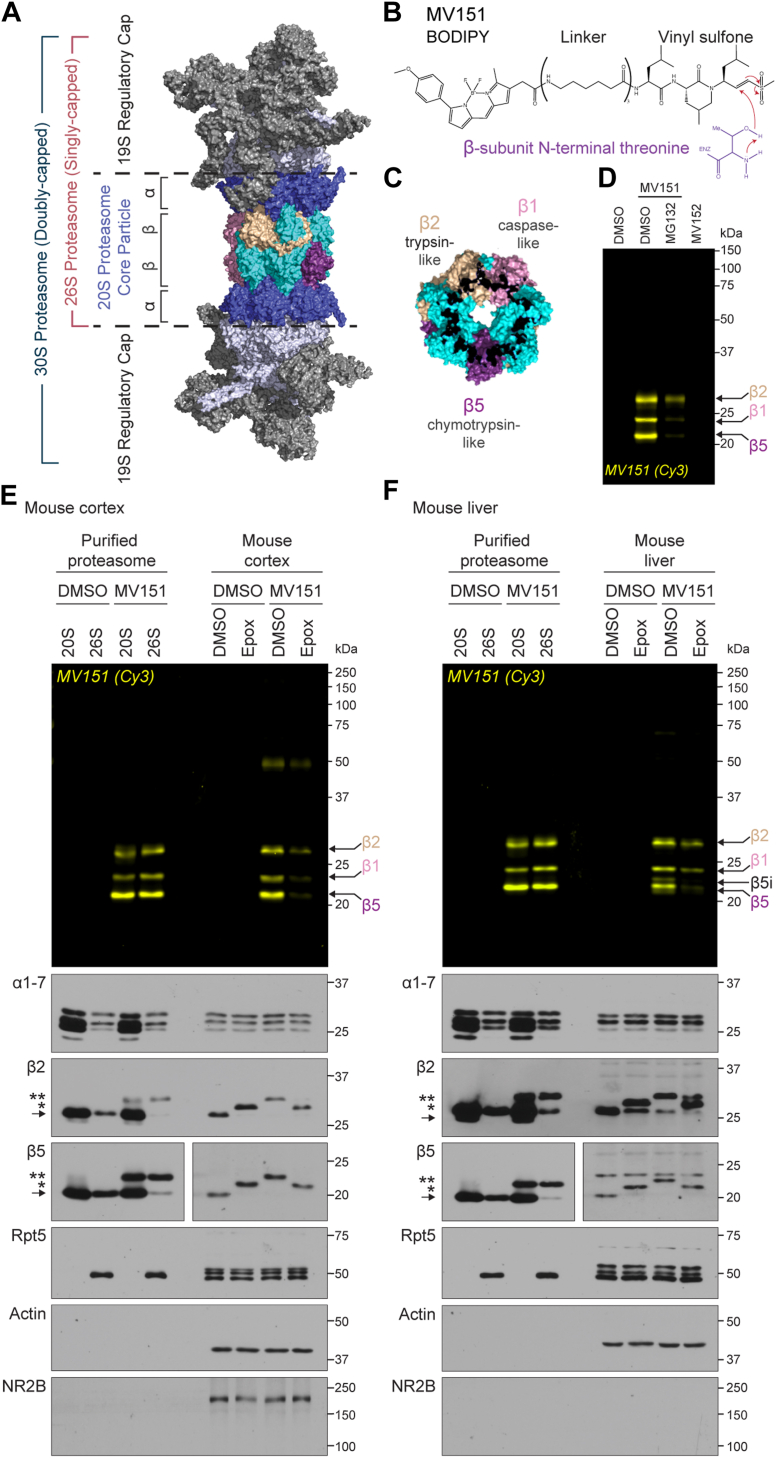
**MV151 is a robust tool for detecting β-subunit activity in purified proteasomes and proteasomes from mouse brain and liver tissue.***A*, structure of the human proteasome (PDB: 5GJR). Modified from Huang *et al.* (46) and adapted from PyMOL. *B*, chemical structure of the activity-based probe MV151. Reaction with the N-terminal threonine (*purple*) of the catalytically active proteasome β subunits is shown. *C*, structure of the proteasome β ring with highlighted catalytically active β1 (*pink*), β2 (*gold*), and β5 (*purple*) subunits (PDB: 5GJR). Modified from Huang *et al.* (46). *D*, purified human 20S proteasome was incubated with vehicle (DMSO), MV151, or an inactive form of MV151 (MV152). MV151-treated samples were incubated with vehicle or proteasome inhibitor MG132. Samples were applied to SDS-PAGE and imaged with the Cy3 channel to detect MV151 labeling. MV151 bands correspond to individual catalytically active β subunits (*arrows*). *E*, *top*, purified human 20S/26S proteasomes or lysates from mouse cortical tissue were incubated with vehicle (DMSO) or MV151. Each cortical sample was also incubated with vehicle (DMSO) or the proteasome inhibitor epoxomicin (Epox). All samples were applied to SDS-PAGE and imaged with the Cy3 channel to detect MV151 labeling. *Bottom*, MV151 gels were immunoblotted with antibodies to the indicated proteasome core subunits (α1–7, β2, β5), a 19S regulatory particle subunit (Rpt5/S6), actin, or neuronal-specific NR2B. β2 and β5 immunoblots show unmodified subunit (*arrow*), ∼0.5 kDa epoxomicin-shifted subunit (∗), and ∼1.1 kDa MV151-shifted subunit (∗∗). *F*, *top*, Cy3 in-gel visualization of MV151 following treatment of purified human 20S/26S proteasome or lysates from mouse liver tissue as in (*E*). *Bottom*, representative immunoblots with indicated antibodies following treatment of samples as in (*E*). Note the presence of catalytically active inducible β subunit (β5i). See also Fig. S1.

Peptide-based model substrates have been used to detect proteasome activity for several decades (61, 62). These substrates are mostly composed of a 3 to 4 amino acid sequence specific for individual catalytically active proteasome β subunits (β1, β2, β5). The target peptide sequence is attached to a fluorescent or bioluminescent reporter. The most widely used fluorescent substrate reporter is 7-amino-4-methylcoumarin (AMC) (61, 63). AMC is attached to distinct peptide sequences to generate substrates for individual subunits: β5 (Suc-Leu-Leu-Val-Tyr-AMC; Suc-LLVY-AMC), β1 (Z-Leu-Leu-Glu-AMC), and β2 (Boc-Leu-Arg-Arg-AMC) (64, 65). Proteasome cleavage of the substrate uncages the reporter, and the fluorescent signal increases proportionally to the amount of substrate cleaved. This cleavage event depends on the AMC substrates having access to the inside of the 20S proteasome. Once inside the 20S proteasome, the catalytically active β subunits cleave these peptides. The enhanced opening of the 20S proteasome, to allow for AMC or any substrate to enter, can be accomplished by the presence of a 19S cap (66, 67, 68), activators (69, 70), fatty acids (71, 72), polylysine (73), cardiolipin (74), polycations (75), sodium dodecyl sulfate (76, 77), hydrophobic peptide analogs (63, 78) or mutation of α3 subunit (79, 80).

Despite widespread use, several features of AMC substrates limit their utility in measuring proteasome activity: (1) The peptide-based substrates are not cell-permeable, limiting the use of this technique to purified proteasomes or cell/tissue lysates. (2) While there are different AMC substrates for distinct catalytically active β subunits, the peptide substrates that are designed for β1 or β2 activities require more optimization and are not as efficient as the ones specific for β5 (81, 82). (3) Most studies investigating the changes in proteasome activity in disease states use the AMC fluorescent signal in a plate reader assay as a readout for proteasome activity in a crude cell or tissue homogenate. The non-specific cleavage of the substrate might be induced by other proteases in the crude biological samples, potentially producing high background or false-positive signal (38, 82, 83, 84, 85). Moreover, these plate reader assays provide no details with regard to different proteasome complexes that are actively degrading the substrate. (4) The hydrophobic nature of the AMC substrates has been documented to artificially modulate the activity of distinct subunits above concentrations of 40 to 50 μM through the interaction of noncatalytic residues of the proteasome core particle (63, 72, 86). This interaction has been shown to participate in the opening of the 20S proteasome without the 19S cap and, depending on the specific AMC substrate, activation or inhibition of individual β subunits (63, 87). The precise mechanism for these effects remains to be fully delineated. In human AD brain studies, the catalytic activity of the proteasome has almost exclusively been measured with AMC substrate concentrations above 50 μM in fluorescent plate-reader assays (84, 88, 89, 90, 91, 92). At these higher concentrations, the 20S proteasome would be activated independent of any existing 19S cap or activator (63). Thus, conclusions about whether changes in the proteasome in the AD brain are relevant to 19S-capped or uncapped 20S proteasome remain in question. To overcome several limitations of using AMC and fluorescent plate-reader assay, it is essential to couple these studies with native-gel detection of the intact proteasome complexes using the AMC substrates (93).

Due to the aforementioned limitations of AMC peptide-based model substrates, several groups have developed activity-based probes (ABPs) that bind covalently with unique specificity to the N-terminal threonine active sites of proteasome β subunits (caspase-like (β1); trypsin-like (β2); chymotrypsin-like (β5)), enabling time-resolved and quantitative measurements of available proteasome subunit activity *in vitro* and *in vivo* (94, 95, 96, 97, 98, 99) (Fig. 1*B*). Unlike peptide-based model substrates, which detect *in vitro* model substrate degradation by active proteases in a given sample, ABPs allow for specific and covalent labeling of the active proteasome subunits in cell and *in vivo*. While this covalent association of ABPs prevents further degradation by the labeled proteasome, the amount of binding is proportional to the relative openness of the 20S proteasome and the individual β subunit activity. Despite the development of these robust tools, they remain underutilized in mammalian neuroscience, human neurodegeneration studies, and proteasome studies across other tissues.

Proteasome-specific ABPs are directed to their targets only through the open-gated 20S proteasome rather than random diffusion (100, 101). If the gate is closed or the active β subunits are occluded/modified by other compounds or molecules, the probe cannot bind to the proteasome, thus the name “activity-based” probes (96, 98, 100). MV151 (BODIPY(TMR)-Ahx3-L3-VS) is a fluorescent, cell-permeable ABP that enables labeling of the active proteasomes in lysate, whole cells, and *in vivo* (96, 98, 99, 100, 102). The vinyl sulfone reactive group on the C-terminus of MV151 undergoes a nucleophilic attack by the N-terminal threonine residue of inducible or constitutively active proteasome β1, β2, or β5 subunits (Fig. 1, *B* and *C*). Because MV151 covalently and irreversibly labels all inducible or constitutive catalytically active β subunits, it is an invaluable tool for profiling the complete activity state of all proteasome complexes and individual subunits within cells, tissues, or *in vitro* (95, 103, 104).

The use of ABPs in human samples has been limited, so we considered that their optimization would better define proteasome activity changes and broaden their use in the field of neuroscience. Here, we optimized the application of multiple proteasome tools to study the proteasome composition, abundance, and available activity using purified proteasome, mouse brain tissue, mouse liver tissue, and primary neuronal cultures. Information from these studies was then applied to our efforts to study proteasome biology in human AD patient brains. Through our experimentation, we revealed that ABPs provide a powerful, robust, and reproducible approach to evaluating proteasome biology across neuronal tissue samples and in cells. We determined that using these tools can provide details related to the overall available activity of various proteasome complexes and their individual β subunits. This led to the observation that, consistent with reports in cancer cells, neurons retain an abundance of accessible and active 20S proteasome complexes without the 19S regulatory particle. We believe this to be a vital observation as it changes our perspective of ubiquitin-independent proteasome function in neuronal biology. Applying these approaches to frozen human brain tissue, we were surprised to find that the 20S proteasome without 19S cap was the only measurable and accessible active proteasome. Subsequently, we revealed a significant increase in available 20S proteasome activity in severe cases of AD. This finding is in contrast to previous studies and provides a foundation for future studies linking proteasomes to the progression of AD. Taken together, our study provides a framework for standardizing the investigation of proteasomes in the nervous system and reveals unexpected insights into changes in proteasome composition, abundance, and activity in the aging human brain.

## Results

### MV151 is a robust tool for detecting endogenous β-subunit activity in all proteasomes from mouse brain tissue

**Supplemental Figure S1:**
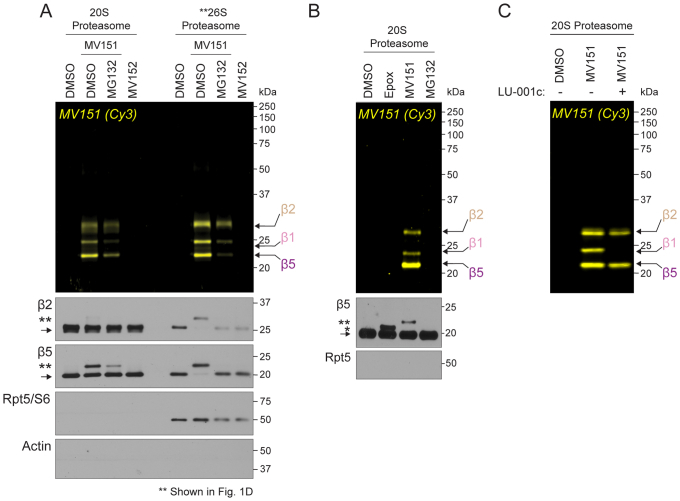


The activity-based probe MV151 covalently associates with proteasome catalytic β subunits and has a fluorescent reporter tag that enables in-gel fluorescence detection of individual β subunits from an intact proteasome complex (96) (Fig. 1, *A*–*C*). To use MV151, we incubated 0.25 μg of purified human erythrocyte 19S-capped 20S proteasome (26S) with 0.5 μM MV151 for 1 h at 37 °C in the presence of vehicle (1% DMSO) or the reversible proteasome inhibitor MG132. Samples were prepared for SDS-PAGE, and the gel was imaged with a Typhoon Imager using the Cy3 channel (excitation: 532 nm). In this gel, we detected fluorescence at 23, 24, and 27.6 kDa, consistent with the labeling of the specific core proteasome β subunits (β5, β1, β2, respectively) covalently bound with MV151 (1.1 kDa) (Fig. 1*D*). We observed no fluorescence in lanes containing samples without MV151 treatment and reduced fluorescence in lanes with samples treated with MV151 in the presence of MG132 (Fig. 1*D*). To further verify our MV151 signal, we used a control compound MV152, in which the vinyl sulfone group has been reduced to an ethyl sulfone, thus eliminating the binding of this probe to the active sites of proteasome subunits. Despite MV152 containing a fluorescent reporter, we detected no signal in lanes containing samples treated with MV152 (Fig. 1*D*). These data are consistent with previous studies and confirm the specificity of MV151 labeling of active β subunits within a purified 26S proteasome complex (96, 105). Similar results were observed when using purified human erythrocyte 20S proteasome without the 19S cap (20S) (Fig. S1*A*).

MV151 has not been widely used in the field of neuroscience to measure proteasome activity within mammalian brain tissue. We collected adult mouse cortical tissue to begin to optimize MV151 use in mammalian brain tissue samples. We prepared a homogenate followed by incubation of 20 μg of tissue extract with 0.5 μM MV151 for 1 h at 37 °C in the presence of vehicle (1% DMSO) or the irreversible proteasome inhibitor epoxomicin. Similar to Figure 1*D*, we detected fluorescence at 23, 24, and 27.5 kDa, consistent with the labeling of the specific core proteasome β subunits (Fig. 1*E*). We also noted that sometimes we observe a minor signal at 50 kDa with samples pretreated with MV151 (Fig. 1*D*, mouse cortex). Based on previous work in other systems, it is likely that this signal is a non-specific cysteine protease (106). In addition, we performed similar experiments using adult mouse liver tissue as a means for comparison between tissues and evaluation of the robustness and reproducibility of our approaches. Similar to brain tissue, in liver tissue samples, we observed no fluorescence in lanes containing samples without MV151 treatment and reduced fluorescence in lanes with samples treated with MV151 in the presence of epoxomicin (Fig. 1, *E* and *F*). As controls, we included purified human 20S and 26S proteasomes (Fig. 1, *E* and *F*, left side). We observed that the MV151 signal was fairly even across each active subunit in brain and liver lysate samples. We took this to mean that proteasomes in mouse and liver tissue lysate are open and have active β subunits. As discussed earlier, the entry of ABP substrate into the 20S proteasome in these lysates could be accomplished by the presence of a 19S cap (66, 67, 68), activators (69, 70), fatty acids (71, 72), cardiolipin (74), hydrophobic peptides (63, 78), or possibly other unknown cellular factors such as polyamines.

Imaged gels were then prepared for immunoblot analysis using antibodies raised against the proteasome core subunits α1, 2, 3, 4, 5, 6, 7, β2, and β5. As a set of loading controls, we used antibodies raised against a 19S regulatory particle subunit (Rpt5/S6), actin, and the neuronal receptor N-methyl D-aspartate receptor subtype 2B (NR2B) (Fig. 1, *E* and *F*). We observed that in lanes with samples treated with MV151 (∼1.1 kDa), the catalytic subunits β2 and β5 signals showed the presence of a higher molecular weight (MW) band indicative of MV151-associated (∼1.1 kDa) subunits (denoted with ∗∗ in the figures). Throughout the article, we will keep referring to this MV151-bound active subunit population in our immunoblots as the “shifted band” (Fig. 1, *E* and *F*). As expected, we did not observe the shifted band in non-catalytic α-subunits. MV151 was not able to completely shift all β5 or β2 of purified 20S proteasomes, consistent with a portion of the purified 20S proteasomes being inactive (Figs. 1, *E* and *F* and S1, *A* and *B*). Interestingly, samples treated with the covalent pan-proteasome inhibitor epoxomicin (∼500 Da) showed a 500 Da shifted band (denoted with ∗ in the figures). When both MV151 and epoxomicin were combined in these lysates, we found that epoxomicin outcompetes MV151, as indicated by the presence of only the 500 Da epoxomicin shifted band (Fig. 1, *E* and *F*). The treatment of the purified proteasome with epoxomicin and MV151 led to similar results (Fig. S1*B*).

Despite considerable effort, we were unable to find a reliable commercially available antibody raised against β1 subunit to measure the β1 band shift. To validate that our MV151 signal at the correct molecular weight of β1 was indeed β1, we used a β1 specific irreversible, covalent inhibitor, LU-001c (97). LU-001c has an epoxyketone warhead instead of a vinyl sulfone (MV151). The epoxyketone undergoes a nucleophilic attack by the N-terminal active threonine residue on the β1 subunit, leading to a covalent bond and irreversible proteasome inhibition. The mechanism of action of LU-001c is actually very similar to MV151. However, in LU-001c, next to the warhead, there is a recognition site specific for β1 subunit, enabling the β1 subunit specificity. We determined that the introduction of LU-001c into our assays with the purified 20S proteasome completely blocked the association of MV151 with the band at the corresponding size of the β1 subunit (Fig. S1*C*).

Our observations that all β2 and β5 signals showed the shifted band in mouse cortex and liver homogenates with MV151 or epoxomicin led us to conclude that all proteasomes (*e.g.*, 30S/26S/20S or alternative 20S complexes) in the healthy adult mouse cortex and liver must, to some capacity, be open, active and in an intact complex. This was in contrast to the purified 20S proteasome mixture, which had a portion of the proteasome complexes that appeared to be inactive and thus cannot bind the MV151 probe. Considering all of our initial observations with MV151, we were next curious as to the kinetics of the β subunit activities and whether we could detect differences in the activity of distinct β subunits across tissues using the ABP.

### Kinetics of proteasome catalytic β subunits are measurable using MV151 across tissues homogenates and in neuronal cell culture

**Figure 2:**
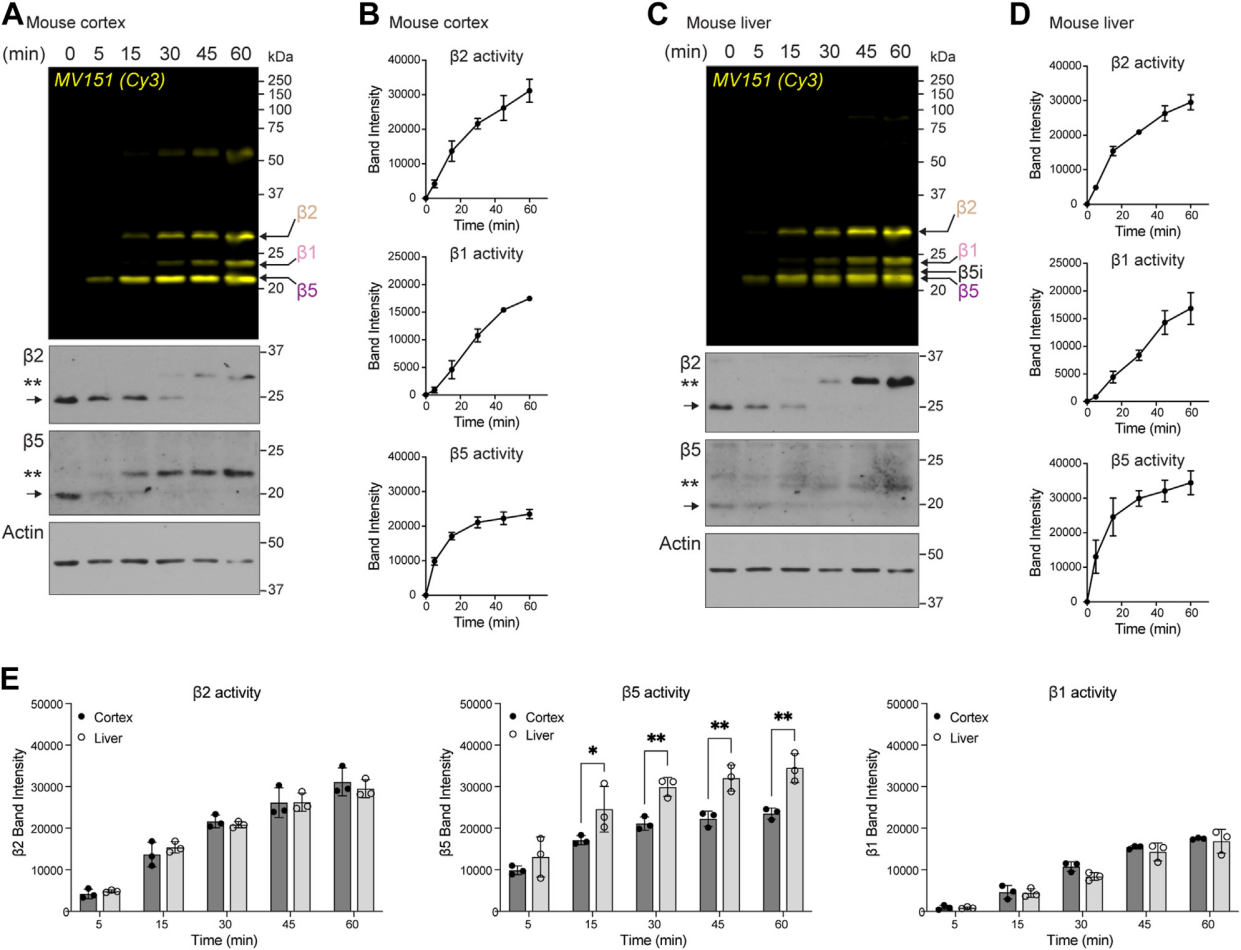
**The kinetics of proteasome catalytic β subunits are measurable using MV151 across tissue homogenates.***A*, *top*, lysates from mouse cortical tissue were incubated with MV151, and samples were collected at indicated time points. Samples were applied to SDS-PAGE and imaged with the Cy3 channel to detect MV151 labeling. MV151 bands correspond to individual catalytically active β subunits (*arrows*). *Bottom*, MV151 gels were immunoblotted with antibodies to the indicated core proteasome subunits (β2, β5) and actin. β2 and β5 immunoblots show unmodified subunit (*arrow*) and ∼1.1 kDa MV151-shifted subunit (∗∗). *B*, quantification of actin-normalized MV151 signals from (*A*). Data points are represented as mean ± SEM (n = 3). *C*, *top*, Cy3 in-gel visualization of MV151 time course with lysates from mouse liver tissue treated as in (*A*). *Bottom*, representative immunoblots of the MV151 gel as in (*A*). *D*, quantification of actin-normalized MV151 signals from (*C*). Data points are represented as mean ± SEM (n = 3). *E*, comparison of quantification of MV151 signal from mouse cortex (*B*) and mouse liver (*D*). Data are represented as mean ± SEM (n = 3). Statistical test: two-way ANOVA (∗*p* < 0.0332, ∗∗*p* < 0.0021). See also Fig. S2.

It has been shown that MV151 has a higher specificity for the active β5 subunit compared to β1 and β2 (98). Whether this is true for all proteasomes across tissues remains unclear and has never been addressed in the nervous system. Having optimized the use of MV151 in the mouse tissue, we performed a time course experiment with MV151 to better understand the kinetics of MV151 incorporation into individual β subunits across different tissue types. Briefly, mouse cortical or liver tissue homogenate was incubated with MV151, and at various times, samples were collected for analysis. Samples were prepared for SDS-PAGE and fluorescence imaging to measure relative amounts of MV151 incorporation over time into each of the catalytically active β subunits (Fig. 2*A*). We performed the same time course experiment with mouse liver tissue homogenates (Fig. 2*C*). Imaged gels were then prepared for immunoblot analysis using antibodies for the proteasome core subunits. Changes in the kinetics of MV151 incorporation measured by Cy3 fluorescence across subunits were further validated by analyzing the time-resolved shifted band of β2 and β5 signals observed in these immunoblots in the mouse cortex (Fig. 2, *A* and *C*). We noted that in overexposed immunoblots, we can detect some non-specific signals for individual β subunits at various size ranges including the 50 kDa range. While these bands may be responsible for the observed MV151 signal at 50 kDa, it is unlikely as we do not see any MV151 signal at the other non-specific molecular weights (data not shown). The normalized band intensity of the Cy3 fluorescence signal of distinct subunits was quantified (Fig. 2, *B* and *D*). Consistent with previous results using purified proteasome (98), we observed more rapid incorporation of MV151 into the β5 subunit when compared to β1 and β2 in both cortex and liver tissue samples. This could be due to a higher specificity of MV151 to β5 subunit or to β5 subunit being more available for MV151 binding (*i.e.*, less occupied with endogenous substrates) than other β subunits. In addition, the lower level of maximal MV151 incorporation into the β1 subunit may have to do with the amount of β1 activity or available β1 for binding to MV151.

When we compared the MV151 labeling kinetics of individual β subunits between mouse cortex and liver tissue, we observed that the β5 subunit from liver proteasomes had a significantly higher overall association of MV151 when compared to that of brain proteasomes (Fig. 2*E*). This is indicative of proteasomes in the liver having more available β5 subunits, while available β2 and β1 subunit activities were relatively similar in brain and liver tissue. While there are several possible explanations for this observation, these data are consistent with previous findings using specific AMC peptides and plate reader fluorescent assays (107). One significant difference between our observations and this previous study is that in addition to β5, Van Kaer *et al.* also observed an increase in β1 and β2 activities. These previous studies are done with individual AMC probes with varying degrees of comparability and are not able to measure the activity of all catalytically active β subunits simultaneously with the same probe. MV151 provides the advantage of being able to simultaneously compare the relative available activities of each catalytically active β subunit. Despite the pitfalls with AMC probes, we believe our data support MV151 as being similar to that of AMC assays in its ability to measure available proteasome activities consistent with prior standard approaches and providing a similar conclusion that different tissues have varying degrees of individual subunit activities.

**Supplemental Figure S2:**
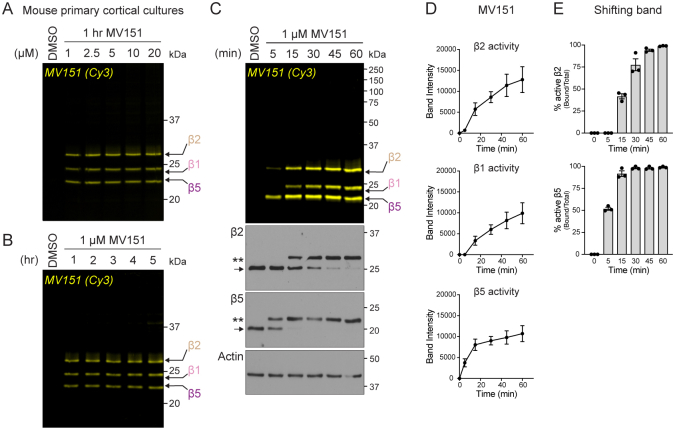


**Supplemental Figure S3:**
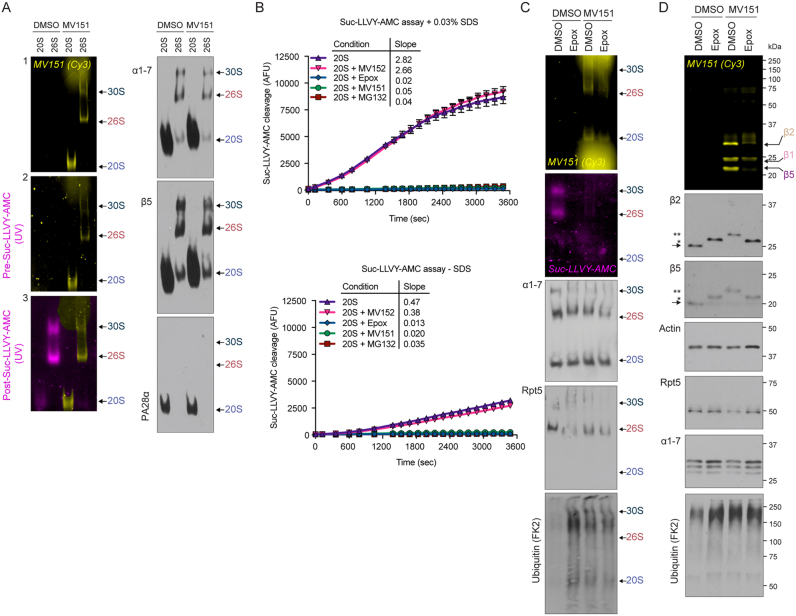


The experiments described earlier were done using homogenates of tissue lysates. To confirm that our data are indeed consistent within live cells, we used our protocols in intact primary mouse neuronal cultures (96, 99). Previous studies had indicated that MV151 needed to be administered for more than 5 h to achieve maximal interactions with endogenous proteasomes in cells (96). Given that MV151 had not previously been tested rigorously in mammalian cortical neurons, we started our inquiry by testing increasing concentrations of MV151. For these experiments, mouse primary neuronal cultures at days *in vitro* (DIV) 12 maintained at 37 °C were treated with MV151. Following treatments, cells were lysed and prepared for either SDS-PAGE (Fig. S2, *A*–*E*) or 4% non-denaturing native gels (Fig. S3, *C* and *D*). We observed that 1 μM MV151 for 1 h of treatment was sufficient to saturate β5, β2, and β1 (Fig. S2, *A* and *B*). Consistent with MV151 fluorescence incorporation and the shifted band of β subunits observed in immunoblots with tissue homogenates, we detected from our neuronal cultures that β5 saturation occurs within 15 min of treatment and shows faster kinetics compared to β2 and β1 (Fig. S2, *C*–*E*). We also noted that the MV151-treated cells labeled all available β5 and β2 subunits and showed equal labeling across each subunit. We took this to mean that all proteasomes in living neuronal cells are readily open and available for degradation. Moreover, inhibition of β5 does not prevent β2 from maximal MV151 binding, consistent with previous reports that blocking the β5 subunit does not impair the activity of β2 (108).

### Activity of 19S-capped and uncapped 20S proteasome complexes are efficiently measured with MV151

Given that we observe all proteasome subunits in neurons to be available and active either using MV151 fluorescence or mobility shift of catalytic β subunits, we predicted that all proteasome complexes within the tissue are also active. Considering that proteasomes can exist in multiple forms, including a 19S-uncapped 20S proteasome, we were curious whether these data indicated that 20S proteasomes are indeed accessible and active in the mouse tissue and to what extent they are active compared to other tissues. By combining the MV151 labeling with native gel approaches, we aimed to look at the relative activities of different proteasome complexes (20S proteasome; 26S singly-capped; and 30S doubly-capped proteasome). Additionally, we used a standard peptide-based model β5 substrate, Suc-LLVY-AMC, to detect the activity of the proteasome complexes for a side-by-side comparison.

**Figure 3:**
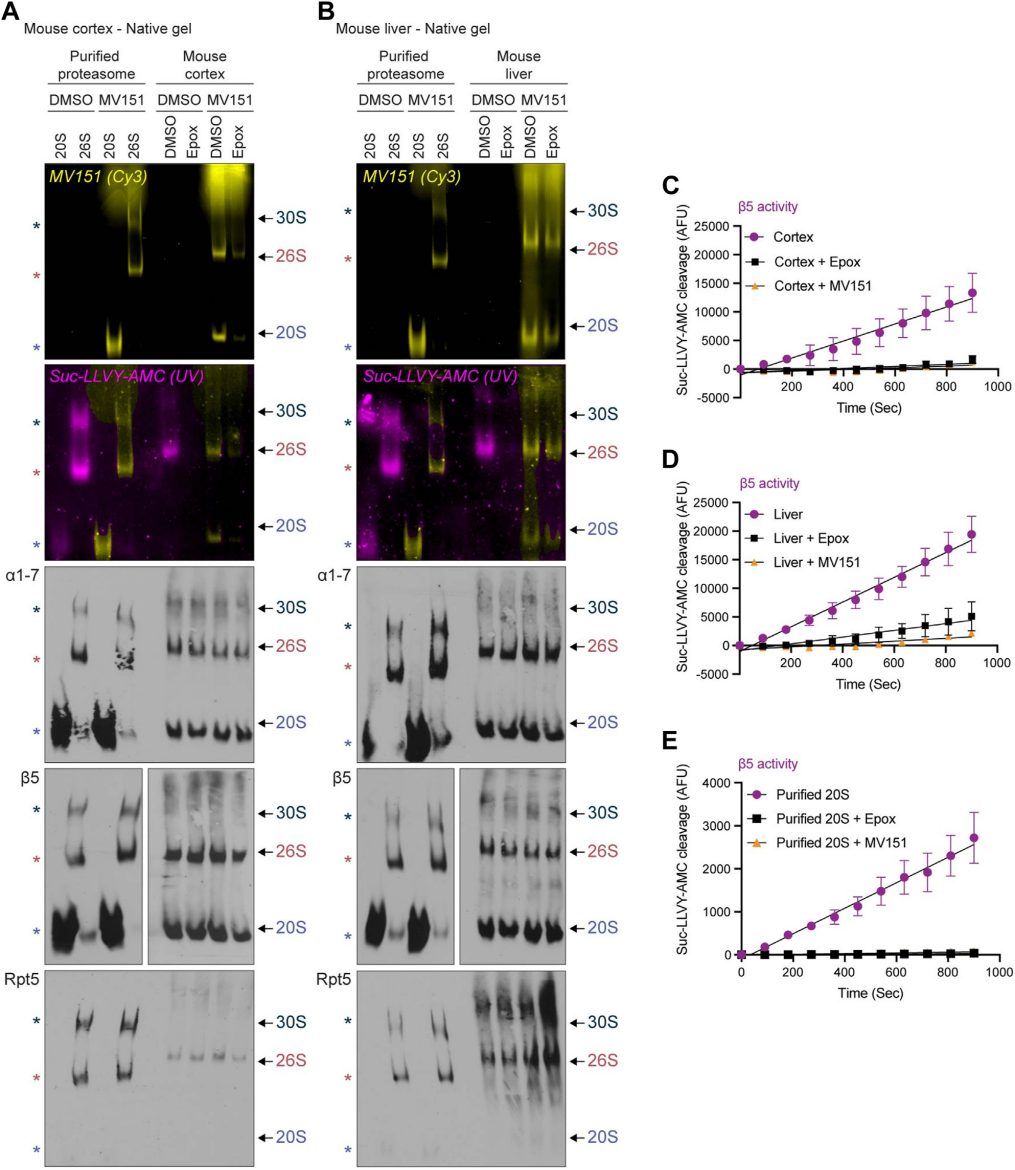
**Activity of doubly-capped (30S), singly-capped (26S), and uncapped (20S) proteasome complexes are more efficiently measured with MV151 than AMC fluorescent substrate.***A*, *top*, purified human 20S/26S proteasomes or lysates from mouse cortical tissue were incubated with vehicle (DMSO) or MV151. Each cortical sample was also incubated with vehicle (DMSO) or the proteasome inhibitor epoxomicin (Epox). To determine the relative activity of distinct proteasome complexes, samples were separated using 4% native-PAGE. First, native gels were imaged with the Cy3 channel to detect MV151 incorporation (*yellow*). Second, native gels were treated with Suc-LLVY-AMC (AMC) and imaged under UV light to monitor proteasome chymotrypsin-like activity (*purple*). *Bottom*, imaged gels were then immunoblotted with antibodies to the indicated core proteasome subunits (α1–7, β2, β5) or a 19S regulatory particle subunit (Rpt5/S6). Cy3, UV, and immunoblot bands correspond to individual proteasome complexes (30S, 26S, 20S) (*asterisks* for purified proteasomes and *arrows* for tissue homogenate). *B*, *top*, in-gel proteasome activity assays with purified human 20S/26S proteasomes or lysates from mouse liver tissue were performed as in (*A*). *Bottom*, representative immunoblots of the native gels. *C*–*E*, Mouse cortex lysates (*C*), liver lysates (*D*), or purified human 20S proteasome (*E*) were treated in a microplate with vehicle (DMSO), MV151, or epoxomicin (Epox), and incubated with Suc-LLVY-AMC. Fluorescence intensity was measured every 1.5 min for 10 min using a microplate fluorescence reader. Data are represented as mean ± SEM (n = 3). See also Fig. S3.

To measure active proteasome complexes, 20 μg cortical or liver tissue homogenate was incubated with MV151 in the presence or absence of epoxomicin. We used purified human 20S or 26S proteasomes as controls. Samples were treated under native conditions and prepared for 4% native-PAGE. We imaged the native gels with the Cy3 channel to observe relative MV151 incorporation into distinct proteasome complexes with and without the 19S cap (20S, 26S, and 30S). MV151 labeling showed a robust signal in the 20S proteasome, suggesting the presence of an open and active 20S proteasome in the case of purified 20S proteasome and cortical or liver tissue homogenate (Fig. 3, *A* and *B*). Following Cy3 imaging, we prepared the gels for in-gel peptide-based substrate treatment with 100 μM Suc-LLVY-AMC, which measures chymotrypsin-like activity using UV detection of the cleaved substrate. As previously mentioned, high concentrations of Suc-LLVY-AMC (>40 μM) have been suggested to be promoting 20S proteasome gate opening. Thus, we tried to use lower concentrations first but were not successful at observing any detectable AMC signal (data not shown). Also, we wanted to use Suc-LLVY-AMC concentrations (∼75–100 μM) commonly used in the literature to reproduce the previous results (88, 89, 90).

To our surprise, we determined that MV151-treated samples showed a signal under UV light which is intended to reveal the AMC signal. This “bleed-through” of the Cy3 MV151 signal at the UV channel was independent of any Suc-LLVY-AMC treatment (Fig. S3*A*). To address this, in our figures, we pseudo-colored (yellow) all the MV151 signal that bled through under the UV channel. While the AMC signal (purple) under UV was detectable and consistent with the MV151 signal (yellow) under Cy3 for 26S and 30S proteasome activity, we detected little to no 20S proteasome-mediated AMC cleavage compared to the MV151 20S proteasome labeling (Fig. S3, *A* and *B*). However, despite the purified 20S proteasome being partially inactive, we noticed that this 20S proteasome showed some AMC cleavage in the native gel. While this was surprising, we believe this is due to the fact that the purified 20S proteasome supplied by Enzo has attached the PA28α activator (Fig. S3*A*).

The binding of MV151 to the 20S proteasome without a 19S cap in lysates indicates that the uncapped proteasome in these lysates was, to some extent, open and active. The lack of in-gel AMC cleavage by these lysate 20S proteasomes could have been due to several reasons: (1) While Suc-LLVY-AMC is specific for one subunit, the MV151 probe can interact with all active β-subunits, thus increasing the total signal intensity and/or (2) the application of the proteasome from lysate into the native gel removes endogenous factors (*e.g.*, hydrophobic peptides or alternative activators) that keep the uncapped 20S proteasome open and active in the lysate. Indeed, the addition of the MV151 to the lysate or cells prior to the application of the sample to native gels may provide the advantage of detecting the intact open and active 20S proteasomes without the 19S cap.

Subsequent to this analysis, we measured the abundance of proteasome complexes in our samples through immunoblotting native gels with antibodies raised against the core proteasome subunits (α1–7, β5) and 19S regulatory cap particle (Rpt5/S6a). Consistent with previous studies, we noticed a high abundance of 20S proteasome complex in the mouse cortex and liver (Fig. 3, *A* and *B*). Taken together with the results from the in-gel AMC assay, there appeared to be a limitation of the Suc-LLVY-AMC in-gel activity assay in the detection of 20S proteasome activity levels, despite there being a significant amount of available 20S proteasome without a 19S cap. Given this limitation, we considered the MV151 signal-to-noise to be superior to AMC in measuring all 20S complex activity and providing a more accurate representation of the cellular environment showing that most, if not all, 20S complexes in cortical or liver tissue lysates and neuronal cells are open and active.

The mechanism for this opening and activity of the 20S proteasomes in lysates of the brain and liver still needs to be clarified. We considered that the 20S proteasome could be opened by alternative caps (PA28αβ or PA200). One may consider that a transient interaction between the 19S-uncapped 20S proteasome with PA28αβ or PA200 would be sufficient to allow for the opening of the barrel and entry of MV151 in lysate and/or in cell. To determine whether the 20S proteasome had alternative caps, we probed mouse brain lysate and purified proteasome with antibodies raised against PA28α and PA200. While it appears in denaturing gels that both PA28αβ and PA200 are expressed in mouse neurons, we were unable to obtain reliable signals from native immunoblots. Unfortunately, the antibodies we used for alternative cap experiments were either not highly specific (PSME4) or produced a high signal band at the bottom of the native membranes, interfering with the proper quantification of alternative cap-associated 20S proteasome (PA28α). It should also be noted that the relative percentage of the alternative-capped proteasome compared to the total proteasome pool under basal conditions is very low for most tissue types, making their detection challenging with native gel immunoblot analysis. We want to highlight that there is a low signal for PA28α-associated 20S proteasome signal in some of our brain tissue samples. While the low percentage of hybrid proteasomes cannot explain the almost complete MV151 labeling of the proteasome, it remains plausible that alternative caps are facilitating gate opening and entry of the MV151. Alternatively, the binding of short hydrophobic peptides to non-catalytic proteasome sites has been shown to induce channel opening in the 20S proteasome, increasing the substrate cleavage (63). Since MV151 and MV152 are composed of the same short hydrophobic peptide sequences in their recognition site, we wanted to confirm that these probes do not artificially promote a peptide-induced increase in 20S proteasome activity. To test this, we examined relative 20S proteasome activity in the presence or absence of MV152 by using plate reader AMC assay (Fig. S3*B*). We showed that MV152 does not cause a peptide-induced increase in 20S proteasome activity, suggesting that the peptide sequence in MV151 and MV152 does not change 20S proteasome activity in our experimental conditions.

We next compared our in-gel assays with the traditional Suc-LLVY-AMC plate reader method, using 75 μM, as an orthogonal approach for measuring the chymotrypsin-like (CT-L) activity of the proteasome in cortical and liver tissue extracts (Fig. 3, *C*–*E*). Supporting our previous findings, we detected more Suc-LLVY-AMC degradation in the liver. Importantly, this assay also showed total inhibition of Suc-LLVY-AMC degradation in the presence of MV151, consistent with MV151 being an inhibitor of proteasome activity and its ability to interact with all available active proteasomes in our sample (Fig. 3, *C*–*E*). In addition, we observed a portion of the AMC signal in our plate reader assay, which was insensitive to proteasome inhibition (Fig. 3, *C*–*E*). While this may be expected, the interpretation of the amount of proteasome being inhibited was more accurate using native gel assays. We also noted that Suc-LLVY-AMC was cleaved by purified 20S proteasome consistent with this proteasome being partially active by the presence of the activator. While supporting our results with MV151 labeling using native and denaturing gels, the plate reader assay was not able to provide details about the composition of the active proteasome complexes or the activities of other catalytically active β subunits. Based on all of our data, we concluded that some portion of the plate reader activity measured using AMC was due to 20S proteasome without a 19S cap which must be evaluated using orthogonal approaches. Given these collective concerns with the AMC plate reader approach, we proceeded to evaluate the mammalian nervous system proteasomes with our combined approaches that rely on ABPs.

The cell permeability of MV151 is an advantage over fluorescent peptide-based substrates. Similar to lysates, when neuronal cell cultures were collected under native conditions, the Suc-LLVY-AMC in-gel assay showed high 30S/26S activity, while MV151-treated cells showed strong probe incorporation with all 20S/26S/30S proteasome complexes (Fig. S3*C*). Additionally, immunoblotting of these native gels with antibodies raised against proteasome core (α1–7) and cap proteins (Rpt5) showed that 30S/26S/20S complexes are highly abundant in neuronal cultures (Fig. S3*C*). To verify our native gel results, samples from these gels were also applied to denaturing conditions. Consistent with our findings in tissue homogenates, as indicated by the shifted bands of catalytically active β subunits, we concluded that all proteasomes (30S/26S/20S) within intact neuronal cells are active (Fig. S3*D*). Because these treatments were done in live cells, we confirmed that the addition of proteasome inhibitors (epoxomicin or MV151) to neurons blocked ubiquitin-dependent degradation and led to elevated signal using antibodies raised against poly-ubiquitinylated proteins. (Fig. S3, *C* and *D*).

From our efforts using purified proteasome complexes, mammalian tissue, and intact primary neuronal cell culture, we have demonstrated the utility of several orthogonal approaches: (1) Native conditions for analysis of proteasome complex (30S/26S/20S) activity and abundance and (2) Denaturing conditions for analysis of all catalytic proteasome β subunit (β1, β2, and β5) activity and kinetics. Given the striking similarity between tissue homogenate and cell assays, we concluded that data from our combined approaches in tissue homogenate are a robust proxy for the composition, activity, and abundance of proteasomes in cells. Thus, we sought to apply these approaches to better understand how proteasomes change in the human brain using tissue homogenates from post-mortem human brain tissue samples.

### Fresh frozen brain tissue from primates or humans with low PMI contains detectable active 19S-capped 20S proteasome complexes

The link between proteasome-dependent protein degradation and human brain disease is still unclear. In particular, there are contradictory findings related to the relative proteasome activity levels in healthy *versus* diseased brains (33, 92, 109, 110, 111). Understanding changes in the proteasome composition, activity, and expression is vital to gaining insight into the role proteasomes may play in human disease as well as building experimental models for future investigation. Thus, we asked whether we could use our established approaches from mammalian brain tissue to more precisely assess the differences in proteasome composition, activity, and abundance in the human brain tissue. To do this, we relied on several brain banks.

**Figure 4:**
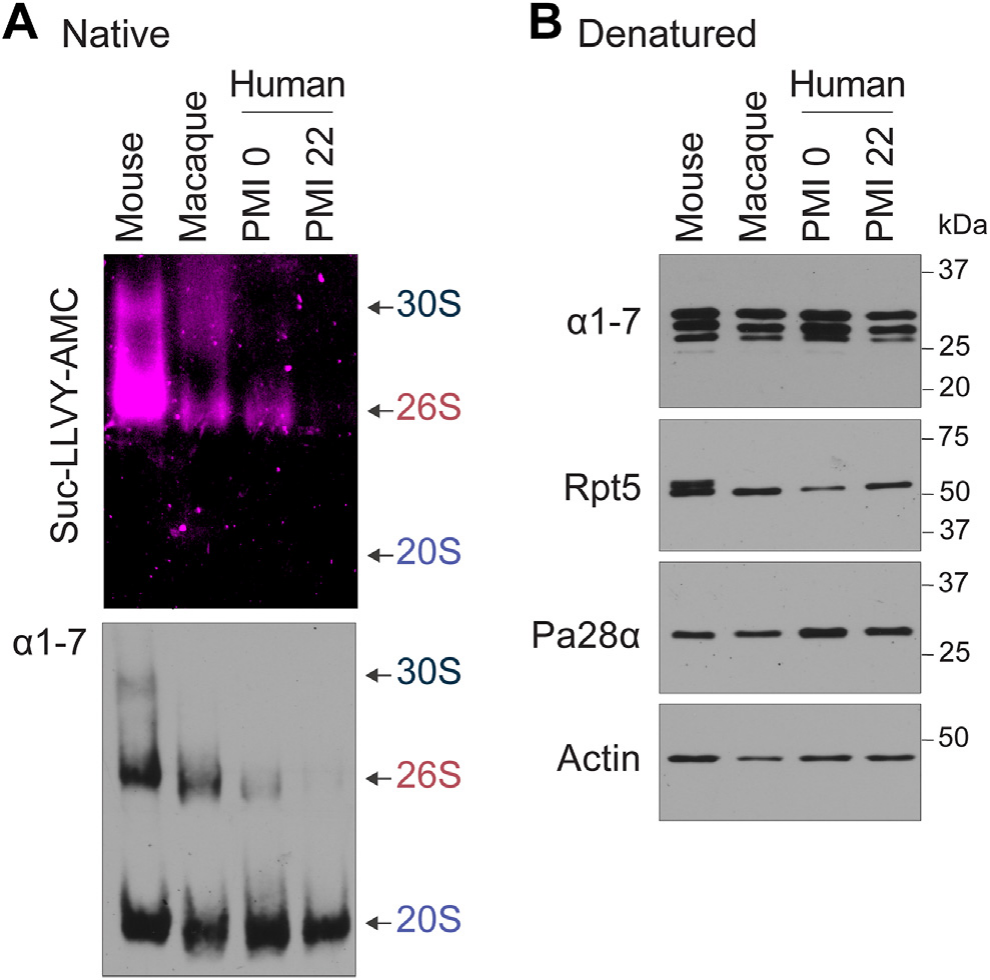
**Human brain tissue contains predominately 19S-uncapped 20S proteasome.***A* and *B*, frozen mouse, macaque, and human brain samples were collected and applied to native (*A*) or denaturing (*B*) gel conditions. For native gels, samples were prepared for AMC assays as previously described, followed by immunoblot analysis using indicated antibodies. For denaturing gels, samples were prepared in denaturing buffer as previously described, followed by SDS-PAGE and immunoblot analysis using indicated antibodies. Detectable capped proteasome signal appears at locations indicated for the 19S-uncapped (20S), singly capped (26S), or doubly capped (30S) proteasome. Note, no AMC signal for the 20S proteasome, no 19S-capped proteasome in PMI 20 h human sample, and significantly reduced capped proteasome for macaque and PMI 0 human brain samples compared to mouse tissue. Also, note equal levels of 20S proteasomes and subunits available in all samples. See also Fig. S4.

**Supplemental Figure S4:**
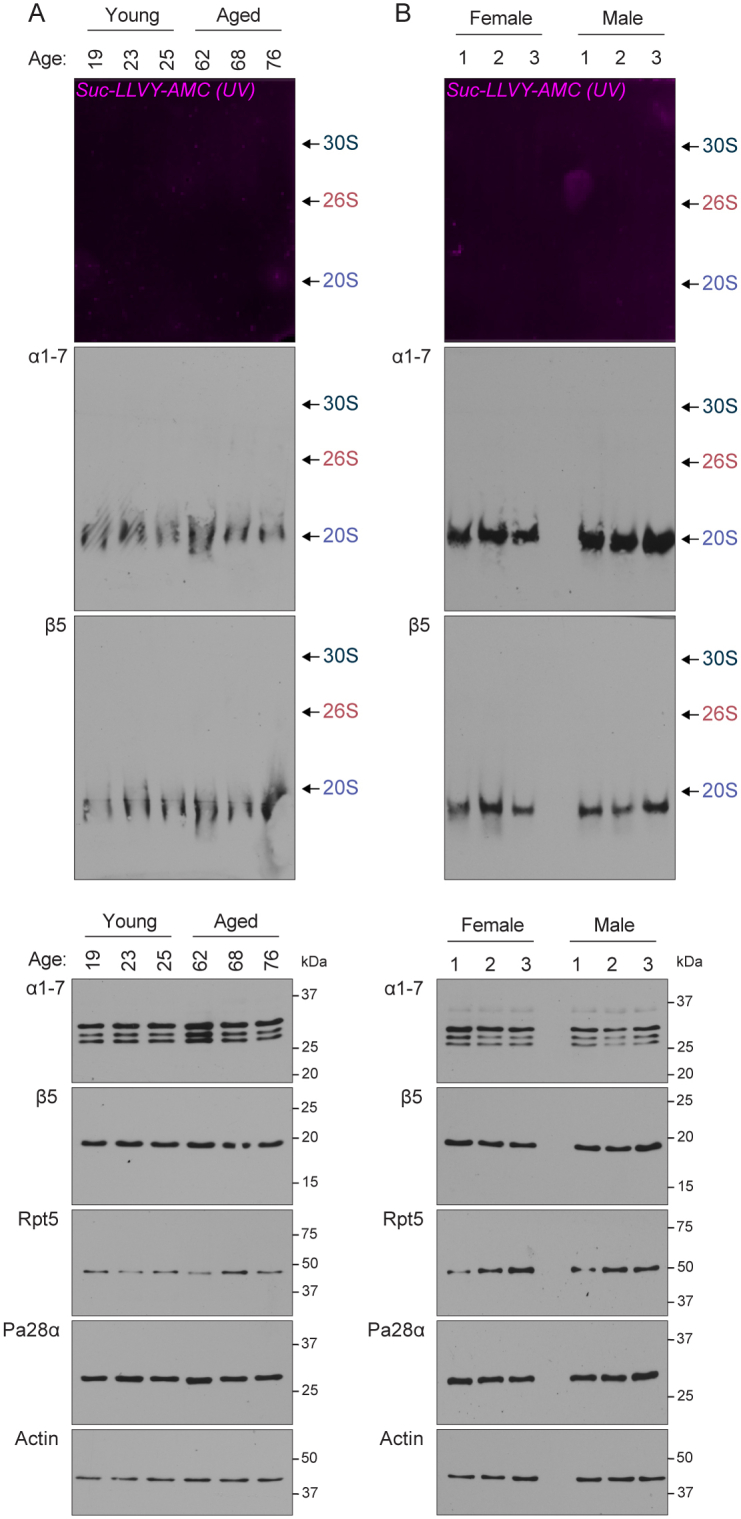


To begin this line of investigation, we compared cortical brain tissues from mice (*Mus musculus*) as well as two primate species, rhesus macaque (*Macaca mulatta*) and humans (*Homo sapiens*). The rhesus macaque tissue was collected immediately following euthanasia and perfusion similar to that of mouse tissue. Human tissue has the caveat of not being collected for a substantial period following death. The majority of human brain tissue has a post-mortem index (PMI) of greater than 20 h (84, 90). To ensure that PMI was not an issue in our studies, we used human biopsy tissue with a low PMI (<2 h) and standard PMI (>20 h) for comparison. To measure active proteasome complexes, 20 μg cortical tissue was treated under native conditions and prepared for 4% native PAGE. Gels were then prepared for in-gel peptide-based substrate treatment with 100 μM Suc-LLVY-AMC. To our surprise, while the AMC signal was detectable in mouse brains for the 19S-capped 20S proteasome, it was significantly reduced in macaque and low-PMI human samples and completely absent in standard-PMI human samples (Fig. 4*A*). Consistent with our results in mouse tissue, there was little to no detection of uncapped 20S activity using Suc-LLVY-AMC. The native gel was then immunoblotted for specific proteasome subunits. Consistent with no capped proteasome signal in standard-PMI human samples, we saw no detectable 26S or 30S proteasome complex (Fig. 4*A*). Similar results from standard PMI human samples were observed regardless of the age or sex of human subjects (Fig. S4, *A* and *B*).

Of the primate samples, only the macaque brain tissue appeared to have a level of 19S-capped proteasome complex that was comparable to mice brain tissue. While one possibility was that the 19S caps were falling off following death, it was curious to note that we did not detect any increase in 20S complexes in any of the primate samples with no capped proteasomes. This indicated that either the capped proteasome was in low abundance in human tissue or that the 19S-capped proteasome complex fell apart after death into its individual components. We reasoned that if the latter was indeed true, then we would expect to see a similar total amount of subunits for the given samples. Indeed, when these same samples were applied to SDS-PAGE and immunoblotted for proteasome subunits and normalized, we did not observe a detectable difference in the amount of individual subunits across all tissue samples (Fig. 4*B*).

**Supplemental Figure S5:**
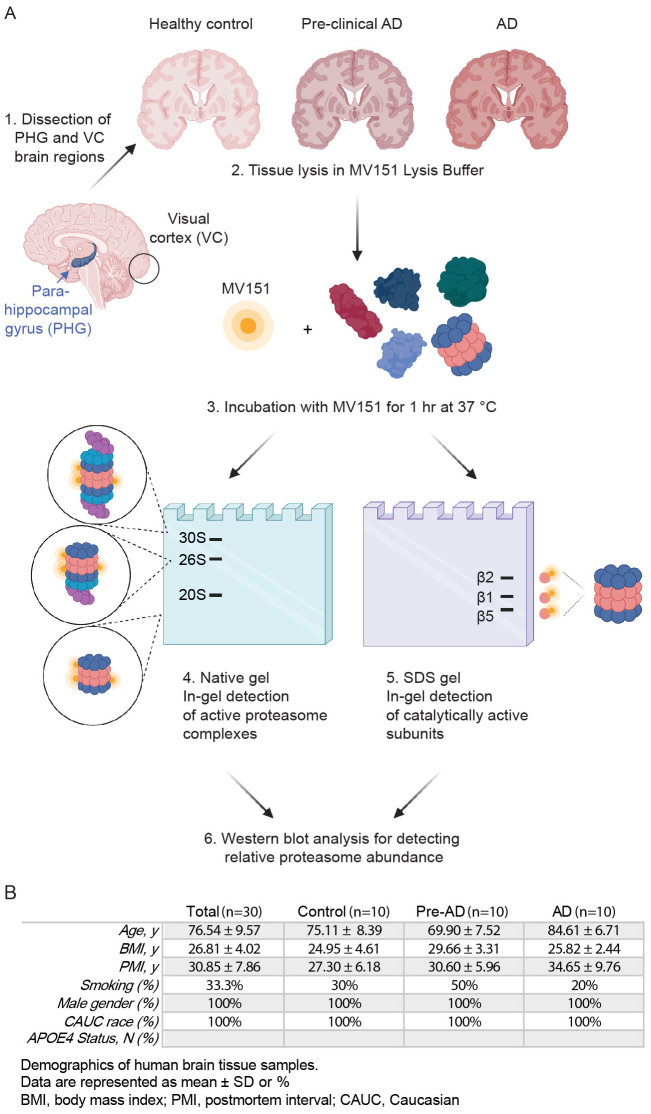


We considered the possibility of proteasome caps getting dissociated during sample preparation; however, the presence of intact 30S/26S purified proteasome in commercially purified samples with little to no free 20S complex and high levels of capped proteasome complexes in mouse brain tissue samples led us to conclude that even if there is a low level of cap dissociation, it would not be to a level of complete removal of caps, as we see in these human brain tissue samples. Another potential explanation for the observation that human brains have little to no 19S-capped 20S we considered is the depletion of ATP following death. However, previous studies have shown that ATP levels and other glycolytic molecules decline similarly in human and mouse brain tissue post-mortem (112, 113, 114). We can detect significant ATP levels in our neuronal cultures while, consistent with these findings, ATP in post-mortem mouse and human tissue is below detection. Because mouse brain tissue and cultures look similar regarding 19S-capped and 19S-uncapped 20S proteasome levels, we believe ATP depletion cannot explain these results. Thus, either due to experimental limitations or unknown biological phenomena, the available 19S-capped proteasome is dramatically different in humans and primates, likely due to the dissociation of the intact complex. However, based on these data, we did conclude that across all mammals, a similar amount of cortical tissue retains roughly equal amounts of proteasome subunits and intact 19S-uncapped 20S proteasome and exists as either a free 20S or is associated with alternative caps. These observations were unexpected and called into question conclusions made in previous studies relying on single assays using similar frozen human brain tissue. Regardless of this caveat, we felt compelled to proceed forward as we are at least able to assess the 19S-uncapped 20S proteasome and potentially evaluate any changes to it across human disease. Because of the limitations of using fluorescent AMC substrates in detecting uncapped 20S proteasome activity, we proceeded forward with the use of MV151 ABP. We chose to use brain tissue from subjects with a standard PMI as there are many more available samples for which we can control several additional and critical parameters (Fig. S5).

**Figure 5:**
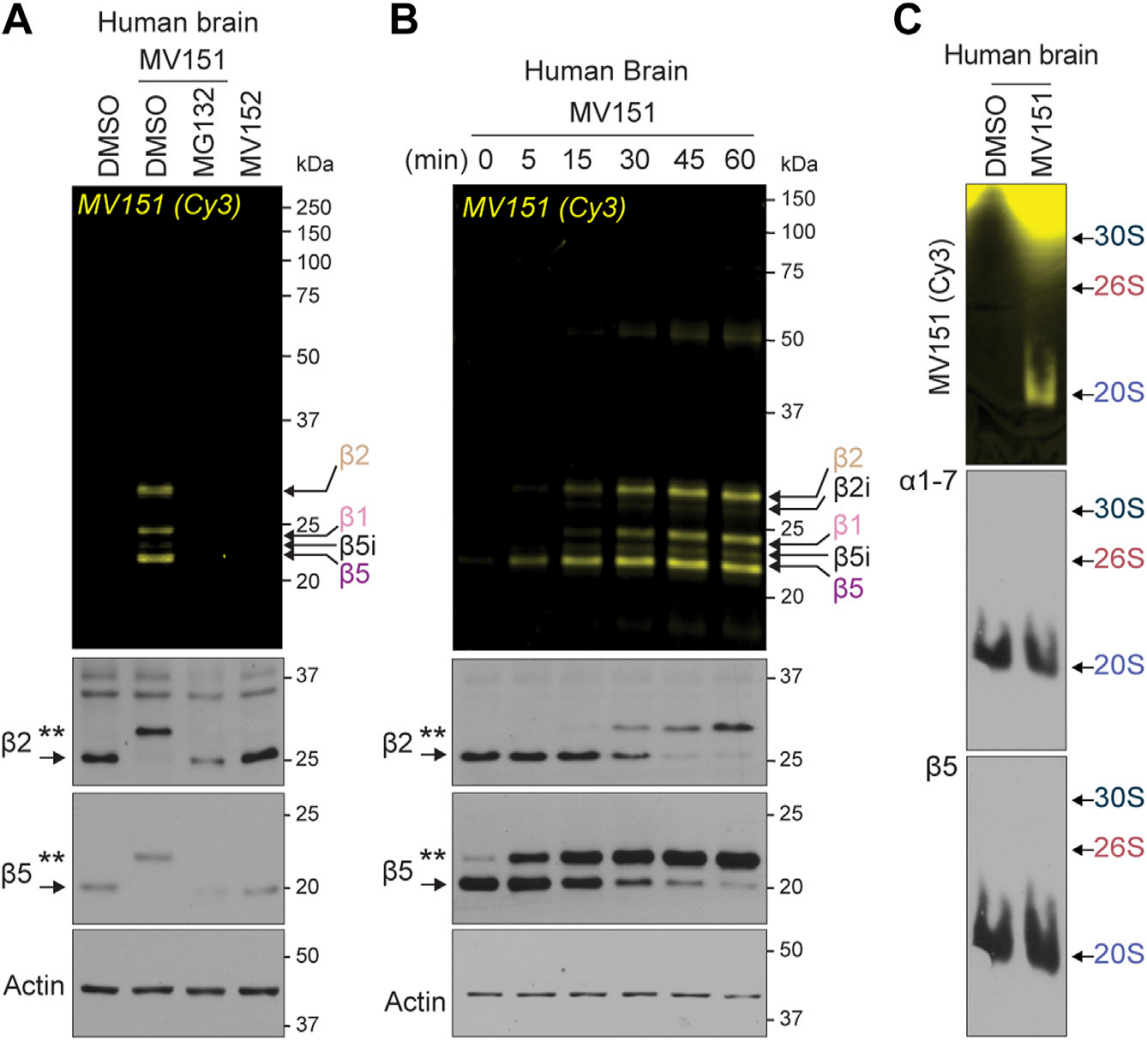
**Human brain tissue contains active 20S proteasome complexes with similar β subunit kinetics to that observed in mice as measured by MV151.***A*, *top*, human brain homogenate was incubated with vehicle (DMSO), MV151, or an inactive form of MV151 (MV152). MV151-treated samples were incubated with vehicle (DMSO) or proteasome inhibitor MG132. Samples were applied to SDS-PAGE and imaged with the Cy3 channel to detect MV151 labeling. MV151 bands correspond to individual catalytically active β subunits (*arrows*). *Bottom*, MV151 gels were immunoblotted with antibodies to the indicated core proteasome subunits (β2, β5) and actin. β2 and β5 blots show unmodified subunits (*arrow*) and ∼1.1 kDa MV151-bound shifted subunits (∗∗). *B*, *top*, human brain homogenate was incubated with MV151, and samples were collected at indicated time points. Samples were applied to SDS-PAGE and imaged with the Cy3 channel to detect MV151 labeling. *Bottom*, MV151 gels were immunoblotted with antibodies to the indicated core proteasome subunits (β2, β5) and actin. β2 and β5 blots show unmodified subunits (*arrow*) and ∼1.1 kDa MV151-bound shifted subunits (∗∗). *C*, *top*, human brain homogenate was incubated with MV151 and processed under native conditions. Samples were applied to 4% native gel and imaged with the Cy3 channel to detect MV151 labeling. *Bottom*, imaged gel was then immunoblotted with antibodies to the indicated core proteasome subunits (α1–7, β5). Cy3 and immunoblot bands correspond to individual proteasome complexes (30S, 26S, 20S) (*arrows*). Note: we only detect the 20S proteasome in human brain tissue. See also Fig. S5.

Consistent with our mouse tissue samples, we were able to show that incubation of the human brain tissue homogenate with MV151 for 1 h at 37 °C labels all three active proteasome subunits. Furthermore, MG132 reduces the incorporation of MV151, suggesting the specificity of MV151 labeling for only active subunits in human brain tissue extracts (Fig. 5*A*). Additionally, the inactive form of MV151, MV152, does not label subunits as shown by in-gel visualization. MV151 labeling was further confirmed by immunoblot with antibodies raised against β2 and β5, which again showed a shifted band (Fig. 5*A*). We also observed similar kinetics of MV151 incorporation into distinct subunits in human brain tissue compared to mouse cortical tissue (Fig. 5*B*). In order to look at the active proteasome complexes, we treated samples under native conditions and imaged the gels with the Cy3 channel (MV151). We detected a high MV151 signal in the 20S proteasome in the human brain tissue. Consistent with our findings in Figure 4, we did not see any 30S/26S MV151 signal (Fig. 5*C*). Again, immunoblot analysis of these native gels showed little to no signal at the 30S/26S size range. Rather, we showed, using immunoblot analysis of the native gels, that most of the proteasome pool was a 20S without a 19S cap (Fig. 5*C*). Notably, the MV151 caused a shifted band in almost all the detectable β2 and β5 signals. This is consistent with our observations that a majority of the active proteasome is 19S-uncapped 20S and the idea that any capped proteasome that falls apart into its individual components only makes up a small fraction of all the proteasome in the human brain. In line with these findings, we found that performing plate reader assays with Suc-LLVY-AMC did not lead to any reliable signal, consistent with there being little to no 30S/26S and the AMC assay being unable to detect 20S activity (data not shown).

### Blinded brain samples from a cohort of healthy controls and patients with AD exhibit 20S proteasome activity that is positively correlated with the severity of the AD state

It is critical to deeply understand the changes in proteasome biology in patients to begin to think about the proteasome’s possible impact on disease progression in humans. After optimizing the experimental conditions and confirming the applicability of the MV151 technique, we developed a workflow for studying the proteasome activity, abundance, and composition in human brain tissue to compare samples from unaffected individuals and those diagnosed with AD (Fig. S5, *A* and *B*). We chose AD as decades of work have indicated that proteasome biology changes in human AD brain tissue. Working with the Lieber Institute for Brain Development (LIBD), we acquired patient samples from two distinct brain regions: the parahippocampal gyrus (PHG) and the visual cortex (VC). The PHG was used as an early onset AD-affected brain region with visible pathological changes, while the VC was used as a control brain region because it is free of neuropathology until Braak stage VI (115, 116, 117, 118). In total, blinded tissue samples from 30 individuals were provided by LIBD (n = 10 healthy controls, n = 10 preclinical AD, n = 10 AD). A summary of sample demographics is provided in Table S1.

**Figure 6:**
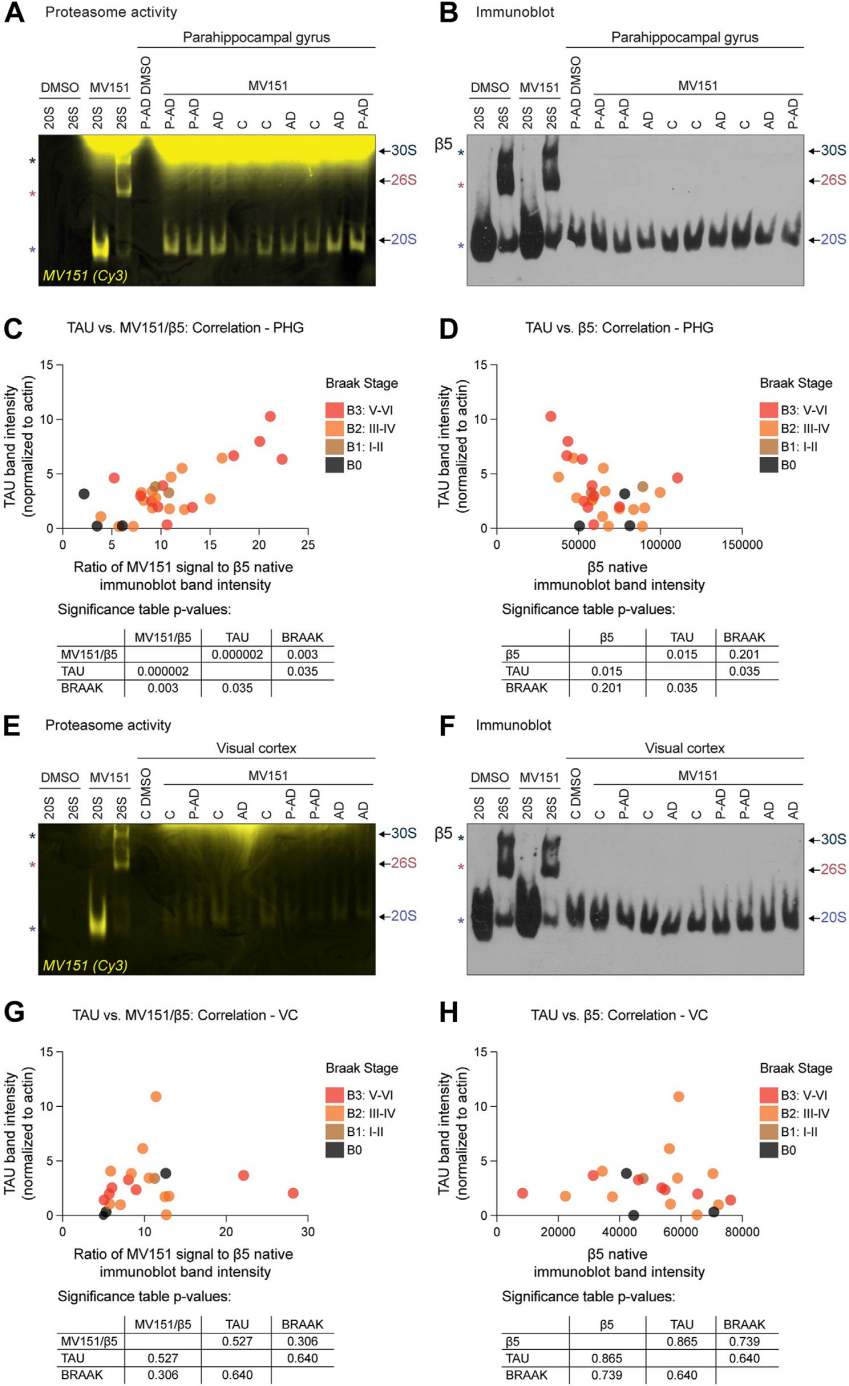
**Blinded brain samples from a cohort of human healthy controls and AD patients exhibit 20S proteasome activity that is positively correlated with the severity of the AD state.***A*, purified human 20/26S proteasomes or tissue lysates from human parahippocampal gyrus (PHG) of unaffected (C), preclinical AD-diagnosed (P-AD), and AD-diagnosed individuals were incubated with MV151. Samples were treated under non-denaturing conditions and run on 4% native gels, which were imaged with the Cy3 channel. *B*, imaged gels were then immunoblotted with antibodies to the indicated core proteasome subunit (β5). Cy3 and immunoblot bands correspond to individual proteasome complexes (30S, 26S, 20S; *asterisks* for purified proteasomes and *arrows* for tissue homogenate). Note: we predominantly detect 20S proteasomes. *C*, multiple variable correlation analysis with Tau band intensity normalized to actin, 20S proteasome MV151 Cy3 signal normalized to β5 band intensity (activity), and Braak Stage as variables in PHG is shown. The significance table shows the two-tailed *p*-value with a confidence interval of 95% as a correlation matrix. *D*, multiple variable correlation analysis with Tau band intensity normalized to actin, 20S proteasome β5 band intensity (abundance), and Braak Stage as variables in PHG is shown. The significance table shows the two-tailed *p*-value with a confidence interval of 95% as a correlation matrix. *E*, Cy3 in-gel visualization of MV151 of purified human 20S/26S proteasomes or tissue lysates from human visual cortex (VC) of unaffected (C), preclinical AD-diagnosed (P-AD), and AD-diagnosed individuals were treated as in (*A*). *F*, imaged gels were then immunoblotted with antibodies to the indicated core proteasome subunit (β5). Cy3 and immunoblot bands correspond to individual proteasome complexes (30S, 26S, 20S) (*arrows*). Note: we predominantly detect 20S proteasomes. *G*, multiple variable correlation analysis with Tau band intensity normalized to actin, 20S proteasome MV151 Cy3 signal normalized to β5 band intensity (activity), and Braak Stage as variables in VC is shown. Statistics of the correlation analysis are shown below the graph. *H*, multiple variable correlation analysis with Tau band intensity normalized to actin, 20S proteasome β5 band intensity (abundance), and Braak Stage as variables in VC is shown with statistics shown below the graph. See also Fig. S6.

**Supplemental Figure S6:**
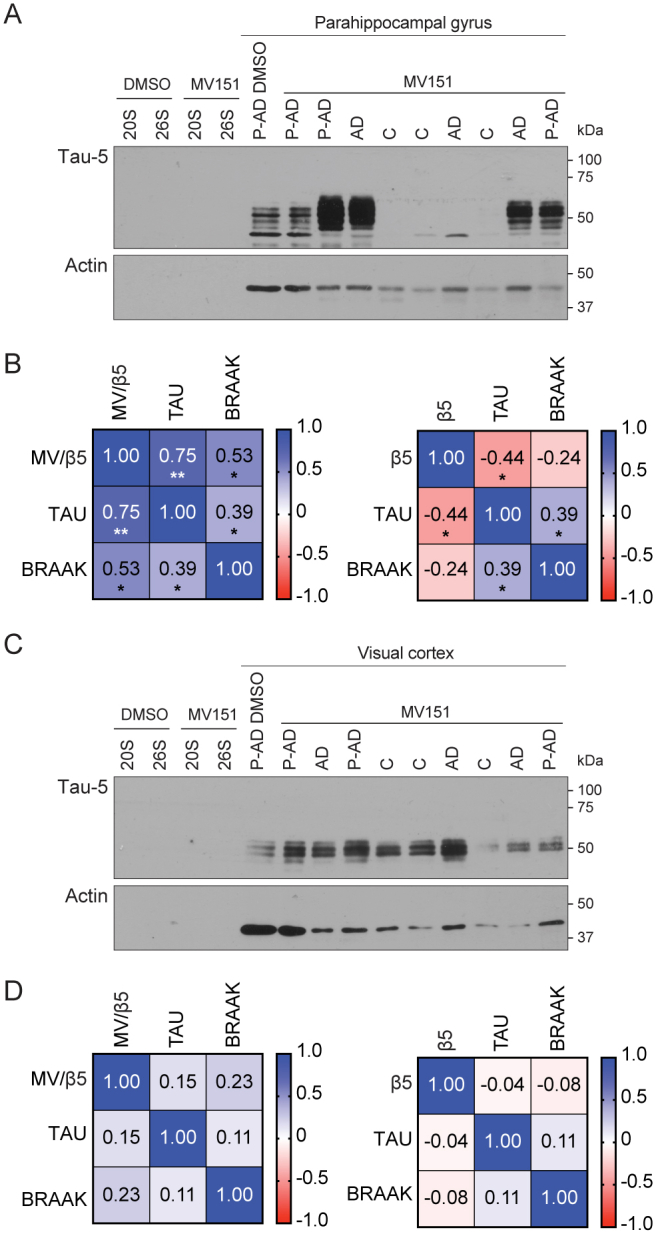


To elucidate a potential link between proteasome activity/abundance and AD state, we incubated MV151 with PHG brain tissue lysates from 30 individuals with different Braak stages and AD pathology. Samples were processed under non-denaturing conditions and run on 4% native gels. In-gel MV151 incorporation was visualized with the Cy3 channel, and the representative gel is shown in Figure 6*A*. Consistent with our findings in Figure 5, we detected MV151 incorporation predominantly into the 20S proteasome complex in these human brain tissue samples. The gels were transferred to PVDF membranes and immunoblots with antibodies raised against β5 were performed to assess the abundance of distinct proteasome complexes (Fig. 6*B*, representative gels). For each of the samples, we also measured Tau levels as a means to normalize the severity of the pathology found in each of these patient samples (Fig. S6*A*). The 20S MV151 band intensity from in-gel visualization and 20S β5 band intensities from immunoblots were quantified. When we performed correlation analysis with Tau levels, normalized 20S MV151 signal, and Braak stage of brain samples, we observed a significant positive correlation between the 20S proteasome activity and the severity of AD state in PHG AD-affected brain region (Fig. 6*C*). Severe AD cases were determined by high Tau levels and Braak stage (V/VI). This result showed that 20S proteasome activity is higher in cases with severe AD compared to healthy human brains. Interestingly, a further novel finding was that there is a negative correlation between the 20S proteasome abundance (20S β5 band intensity) and the severity of AD (high Tau levels and Braak stage) (Fig. 6*D*). Significance tables of the correlation analysis are shown below the graphs. Pearson coefficient values and correlation heatmaps were shown in Fig. S6*B*.

To test whether the changes in 20S proteasome activity/abundance in AD are specific to AD-affected brain regions, we performed the same experiments with the less affected brain region sections (VC) obtained from the same individuals. The 20S proteasome activity (MV151; Fig. 6*E*) and abundance (β5; Fig. 6*F*) were assessed and quantified from all 30 VC tissue samples to determine whether there was any correlation between 20S activity/abundance and severity of AD. We did not detect any significant correlation between Tau levels (Fig. S6, *C* and *D*), Braak stage, and 20S proteasome activity (Fig. 6*G*) or abundance (Fig. 6*H*) in the VC. Pearson coefficient values and correlation heatmaps are shown in Fig. S6, *B* and *D*. Together, the present findings confirm that changes in 20S proteasome activity and abundance in AD are restricted to the AD-affected brain regions (such as PHG) and cannot be observed in less affected brain regions (such as VC).

Taken together, given that human disease, such as AD, is not easily defined by a single parameter (sex, age, Braak stage, Tau, etc.), we feel our blinded analysis of more than 30 subjects more accurately and appropriately demonstrates a correlation between changes in the available activity of 20S proteasome without a 19S cap and the severity of the disease. Our conclusions are in part in contrast to previous studies, which relied on single assay measurements of the proteasome and did not stratify the findings across the multiple parameters associated with each individual human sample. Because of the nature of our approach, it remains possible that 20S proteasomes in lysates from human AD brains are more open and active due to the nature of the environment and/or interaction(s) with various regulators and activators.

### Kinetics of proteasome catalytic β-subunits are distinct in PHG brain tissue from healthy controls compared to patients with AD

**Figure 7:**
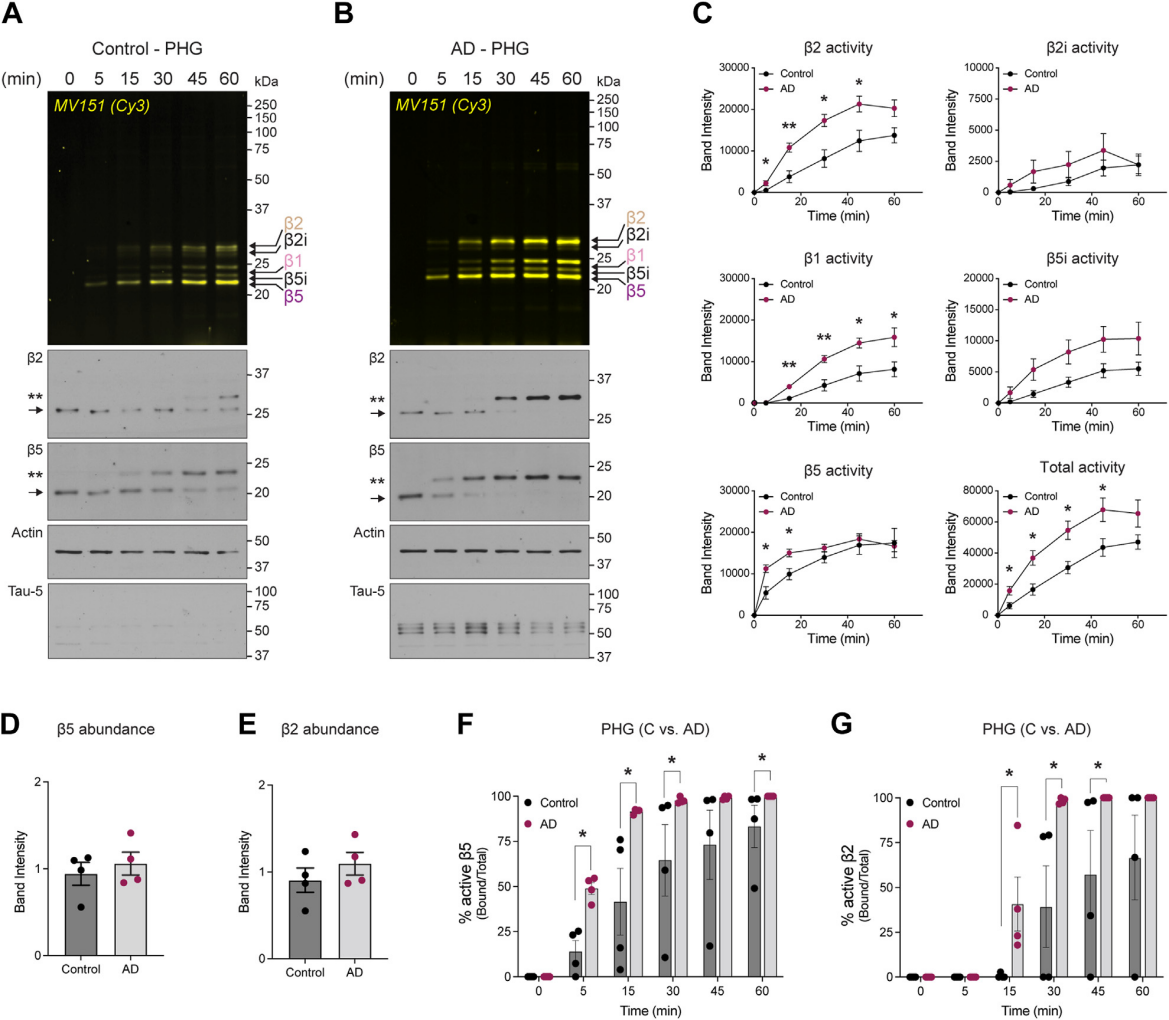
**Kinetics of proteasome catalytic β subunits are distinct in PHG brain tissue from healthy control cases compared to AD patients.***A*, *top*, PHG human brain tissue homogenate from healthy control cases was treated with MV151, and samples were collected at indicated time points. Samples were applied to SDS-PAGE and imaged with the Cy3 channel to detect MV151 labeling. *Bottom*, MV151 gels were immunoblotted with antibodies to the indicated core proteasome subunits (β2, β5), actin, or Tau. β2 and β5 blots show unmodified subunit (*arrow*), ∼0.5 kDa epoxomicin-shifted subunit (∗), and ∼1.1 kDa MV151-shifted subunit (∗∗). MV151 bands correspond to individual catalytically active β subunits (*arrows*). Note: we detect inducible β2 (β2i) and β5 (β5i) subunits. *B*, *top*, Cy3 in-gel visualization of MV151 time course with PHG human brain tissue homogenate from AD-diagnosed cases treated as in (*A*). *Bottom*, MV151 gels were immunoblotted with antibodies to the indicated core proteasome subunits (β2, β5), actin, or Tau. β2 and β5 blots show unmodified subunit (*arrow*) and ∼1.1 kDa MV151-shifted subunit (∗∗). MV151 bands correspond to individual catalytically active β subunits (*arrows*). Note: we detect inducible β2 (β2i) and β5 (β5i) subunits. *C*, densitometric analysis of MV151 Cy3 signal for individual β subunits. Data are represented as mean ± SEM. Statistical test: Student’s unpaired *t* test (∗*p* < 0.05). *D* and *E*, analysis of β5 and β2 protein levels in control and AD cases *via* densitometric analysis of immunoblots. Actin was used as a loading control. Data are represented as mean ± SEM. Statistical test: Student’s unpaired *t* test. *F* and *G*, quantification of % active β5 (*F*) and β2 (*G*) subunits was performed by calculating the ratio of MV151-bound β5 (or β2) band intensity (∗∗) to total β5 (or β2) band intensity in corresponding immunoblots. Data are represented as mean ± SEM. Statistical test: Student’s unpaired *t* test (∗*p* < 0.05). See also Fig. S7.

Observation of elevated 20S proteasome activity led us to question whether the individual proteasome subunit activity might be different in cases with severe AD compared to control samples. Having optimized the use of MV151 in mouse brain tissue (see Fig. 2) to measure the activity of proteasome catalytic β-subunits, we performed a similar experiment with MV151 in human brain tissue homogenates (n = 4 control, n = 4 severe AD). For these experiments, we selected human brain tissue samples that were cases with severe AD (Braak stage V/VI, CERAD 3) and compared them to healthy controls with no Alzheimer’s pathology (AP; CERAD 0, and Braak stage lower than V/IV). Briefly, 20 μg of human brain homogenate from PHG or VC was incubated with MV151, and at various times, samples were collected for analysis. Samples were prepared for SDS-PAGE and fluorescence imaging to measure relative rates of MV151 incorporation over time into each of the catalytically active β subunits in PHG (Fig. 7, *A* and *B*) and VC (Fig. S7, *A* and *B*). Imaged gels were then prepared for immunoblot analysis using antibodies for the proteasome core subunits that detect β2 and β5 of the proteasome. We also used antibodies for actin for quantification normalization and Tau. We noted some decline in Tau signal during the time course, which may be due to degradation; however, this was not observed across all samples, and we observed no degradation of actin. Importantly, we observed that our time course results with MV151-treated human brain tissue mirror the time-resolved band-shifting event of the β2 and β5 signals in our immunoblots in a manner similar to results observed in Fig. S2*C* (Figs. 7, *A* and *B* and S7, *A* and *B*).

In PHG samples, quantifying the normalized band intensity of the MV151 Cy3 fluorescence signal, we observed at most time points across β subunits a significant increase in signal from AD samples compared to control samples from unaffected individuals (Fig. 7*C*). In addition, when we total all the MV151 signals for each individual subunit (β2, β2i, β1, β5i, β5), we observed a significant increase in signal between PHG AD and control samples across time points (Fig. 7*C*). In contrast, despite a trend toward an increase in MV151 signal, similar robust results were not observed when comparing AD and control VC samples (Fig. S7*C*). Interestingly, we found that the inducible β5i subunit was significantly elevated for MV151 incorporation in AD compared to control samples in the VC brain region. Inducible proteasome subunits are indicative of the immune response. These data are consistent with findings that elevated neuroimmune response could occur within the central nervous system and could be a hallmark of age-related neurodegeneration (110).

Similar to MV151 fluorescence changes in PHG, when we quantified the ratio of MV151-bound shifted β5 band (∗∗) to total β5 signal in our immunoblots, we observed a significant increase in the percentage of MV151-bound band signal in AD compared to control samples across time points. Additionally, a significant change in earlier time points indicates faster incorporation of MV151 to β5 subunit (Fig. 7*F*). We believe this to be due to an elevated available 20S proteasome activity as we see that β5 and β2 subunit protein levels do not change in AD compared to control samples (Fig. 7, *D* and *E*). This result was in contrast to our observation in Figure 6 where we see slightly less 20S proteasome in AD *versus* control samples. We believe this discrepancy between these two results may have to do with some residual amount of 19S-capped proteasome that was below detection in our native gel. Although we could not detect any significant increase in β2 subunit activity in AD at 1 h time point, we showed significantly faster MV151 incorporation dynamics at earlier time points in PHG (Fig. 7*G*). Despite a trend toward an increase in band shifting in VC, we did not detect as robust of a significance as in PHG (Fig. S7, *F* and *G*). It is, however, intriguing that VC from patients with AD appears to be developing elevation in proteasome activity which would likely have contributed to the ensuing degeneration of this brain region. Taken together, these data are consistent with the severity of AD brain coinciding with elevated 20S proteasome activity and raise our suspicions about the consequences of this in changing protein degradation pathway in patients with AD. We believe this data to be comprehensive and informative, thus setting the stage for the next steps in thinking about the consequences of these changes to proteasome activity and human brain neurodegeneration.

## Discussion

We have now defined an orthogonal set of biochemical approaches that have the capacity to robustly and reliably provide in-depth details about available proteasome activity in the mammalian nervous system (*i.e.*, mouse primary neuronal cultures, mouse cortical tissue, and human brain tissue). Based on our results, we make five conclusions: (1) ABPs have a high signal-to-noise detection in mammalian neuronal tissue allowing for robust measurements of available proteasome complexes and individual catalytic subunits. (2) ABPs provide a selective advantage in measuring all active proteasome complexes (19S-capped and -uncapped) in mammalian neuronal tissue as they can be applied to intact cells or lysates that retain native conditions while in-gel assays have the caveat of potentially removing critical endogenous factors (*e.g.*, hydrophobic peptides or alternative activators) that keep the native 19S-uncapped 20S proteasome open and active. (3) All proteasomes within mammalian tissue, which includes capped, ubiquitin-dependent 30S/26S, and uncapped or alternatively-capped, ubiquitin-independent 20S, exhibit accessible activity. (4) The dominant form of the measurable proteasome in human brain tissue with standard PMI values is the 20S proteasome. (5) There is a significant elevation in the available activity of 20S proteasomes in the brain of patients with AD. Based on these conclusions, it is clear that the proteasome activity may go beyond ubiquitin-dependent degradation and include, in a major way, ubiquitin-independent degradation *via* the 20S proteasome, which would most certainly give rise to changes in substrate selection, rate of degradation, and products of degradation. While the combined implications of these findings for the mammalian nervous system function remain unknown, this study sets a foundation of approaches to measure proteasome abundance and activity and provides some additional insight into the nature of proteasomes in our brains.

### Approaches to study available proteasome activity

Proteasome activity kinetics has been studied extensively over the past few decades. The majority of these studies have used peptide-based model substrates (84, 85, 119), poly-ubiquitin model substrates (120), or overexpression of fluorescently tagged proteasome substrates (121). The use of these tools has led to great insight into the biophysical properties of predominately ubiquitin-dependent proteasomes *in vitro* and has begun to provide some insight into their activities in cultured cells (83, 122, 123). While peptide-based model substrates have been viewed as the gold standard for measuring proteasome activity, the caveats described earlier limit their use in comprehensively defining the proteasome complexes and activities of individual subunits. In particular, they are not useful in studying proteasome biology in live cells. In plate reader assays, there is very little information that can be established about the makeup of the proteasome complex being measured and the activities of individual subunits. One surprising finding in our work is that while the AMC substrates were not effective at measuring 20S activity, the ABP highlighted that these complexes are open and available for degradation. Several explanations as to why AMC substrates are not efficient at measuring 20S proteasome may have to do with the structure of the AMC, the accumulation of ABP binding *versus* the amount of AMC able to be cleaved, or the sensitivity of the AMC fluorescence *versus* the ABP fluorescence. What is clear is that the ABP efficiently and robustly measures the available activities of all proteasome complexes. Such an observation strongly supports the idea that 20S proteasomes in cells are not latent but rather poised for the active degradation of intracellular substrates. Because all proteasome inhibitors will target any active proteasome in the cell, it is still unclear what the specific role is of one proteasome complex over another. Future efforts to make specific proteasome inhibitors will be of great benefit to such detailed understanding.

Finally, while we did not investigate additional proteasome species that may indicate the association of alternative caps in the native gels in-depth, it remains possible that the activity of 20S uncapped proteasome originates in cells or lysates from alternative cap association. It also remains possible that the nature of molecules in cells (hydrophobic peptides, cations, etc.) could lead to the opening of the 20S proteasome. Regardless of the precise mechanism, it is clear from the ABP data that the 20S core proteasome is open and active. It also appears that within the cell, all available proteasomes are active and likely actively degrading substrates. Future efforts to define the distinct pool of substrates and resulting peptides from individual proteasome complexes will provide a great advance to our understanding of the various activities of proteasomes through the cell and in various states of health and disease.

### Proteasome complexes and neurodegenerative diseases

While several studies have worked toward understanding the nature of proteasome changes in human tissue, little has been done in brain samples from healthy subjects or patients (33, 90, 92). Age-related neurodegenerative diseases (such as AD, Parkinson’s disease, or Huntington’s disease) have long been thought to be impacted in part by changes in proteasome-dependent protein degradation. For decades, many tools and approaches have been applied to address questions related to proteasome function in neurodegenerative diseases (NDs); however, the reason(s) for proteasome dysregulation in the disease state(s) is still unclear. Several studies showed that Aβ directly binds to and competitively inhibits the proteasome, leading to toxic accumulation of Tau and Aβ (124, 125), while other studies suggested proteasome inhibition occurs due to an elevation in oxidative stress in AD (126, 127, 128, 129). Additionally, several groups indicated that the decline in proteasome activity is due to the differential expression of Ubiquitin-Proteasome System (UPS) components in AD, even though the results have been contradictory (91, 92, 123, 124, 130). This is thought to lead to an accumulation of poly-ubiquitylated (poly-Ub) proteins, (92, 124, 131, 132, 133, 134). Several prevailing hypotheses exist to explain the elevation of poly-Ub in AD brains: (1) downregulation of β5 (135); (2) aggregated and accumulated forms of AD-associated proteins (Tau and Aβ) inhibiting proteasome activity (33, 37, 124, 127, 130, 136); and (3) impairment of E3 ligases (such as CHIP, UCHL1, Ube3A, Parkin) in AD (136, 137, 138). In several cases, impairment of E3 ligases has been shown to also directly impact the degradation of Tau, Aβ, and APP, leading to the accumulation of toxic forms of these AD-associated proteins, further disrupting proteasome activity and promoting neurofibrillary tangles (137, 138). While many of these studies have been very informative, for the most part, they have relied on approaches that do not thoroughly and directly evaluate intrinsic features of core proteasome function in the human brain tissue. Based on our results, there is a lack of detectable 26S proteasome activity and abundance in the human brain tissue. This could be due to issues with detection in our assays or an indication that UPS function in the human brain is greatly diminished compared to what is observed in model systems, heterologous cell lines, or iPSC-derived cultures. Further investigation into this would be needed to make such a conclusion.

### 20S proteasome

Despite being described as the latent proteasome, the 20S proteasome activity, independent of the UPS, has been shown to be the main player in proteolysis during oxidative stress (4, 56, 128, 139, 140, 141). Upon oxidative stress, chemical modifications of cellular proteins lead to exposure of hydrophobic residues, which are recognized by the 20S proteasome (142) in an ATP- and ubiquitin-independent manner. The interaction between these exposed hydrophobic residues and 20S α subunits induces a conformational change and opening of the α ring (63). An increase in exposed hydrophobic sites promotes faster protein degradation *in vitro* (143, 144); however, the exact mechanism of oxidation-induced changes in proteolytic activity *in vivo* is still an active area of investigation. A molecular hallmark of neurodegenerative diseases, such as AD and PD, is elevated oxidative damage to proteins in the brain (92, 127, 145). Oxidative stress has been shown to promote UPS inhibition through the impairment of the E1/E2/E3 cascade (146, 147). Consistent with this, inhibiting the UPS does not affect the rate of oxidized protein degradation in intact cells, and purified oxidized proteins are degraded at the same rate when incubated with cell extracts with functional or impaired UPS (141, 148). Based on these studies, our findings of elevated 20S activity in AD tissue might be due to the accumulation of oxidized proteins. However, further investigation is necessary to make conclusions about the link between the observed increase in 20S proteasome activity and oxidative stress in NDs.

In addition to the constitutive proteasome, the immunoproteasome has been shown to be modulated in NDs (110, 149, 150). The immunoproteasome is a proteasome variant with distinct catalytically active subunits (inducible; β1i, β2i, and β5i) formed by pro-inflammatory mediators. The immunoproteasome is critical for proper immune response and more suitable than the constitutive proteasome for the production of peptides for antigen presentation (151, 152). Orre *et al.* (110) showed that Aβ enhances proteasome activity in glial and neuronal cultures as well as in AD mice. They also showed that the elevation in proteasome activity is due to increased immunoproteasome activity in reactive glia surrounding plaques in AD model mice. Other reports showed increased immunoproteasome abundance (135, 153, 154) and activity (155, 156) in AD mouse models due to enhanced neuroinflammation in the diseased brain. Consistent with these studies, we observed an elevated β5i subunit activity in the visual cortex of patients with AD. However, we did not observe similar differences in the PHG of the same patient samples. Our assays cannot differentiate the cell types (neuronal, glial, etc.) since we use whole brain tissue in our assays. The reason for these differences may be due to the differences in disease progression in distinct brain regions. Immunoreactive cells may be more active in VC (*i.e.*, earlier stages of AD) and less affected in later stages. Follow-up studies to test this hypothesis would be of interest in thinking about disease progression.

## Concluding remarks

Decades of work using proteasome inhibitors suggest that the proteasome is vital for neuronal function and survival. As we show in our work, covalent proteasome inhibitors (such as epoxomicin) target all the proteasomes in the brain tissue, and more than 70% of these proteasomes are without the 19S cap. So, the significance of only targeting an uncapped or capped proteasome to neuronal biology has yet to be determined. Even if the proteasome activity is different in the disease state(s), further investigation into the substrate pool of the proteasome, especially the uncapped or alternatively capped 20S, is warranted. It is essential to identify the substrates, modifications responsible for targeting these substrates to the proteasome, and the peptide products generated by the proteasome in the diseased brain. Through the comprehensive protocols and perspective provided in this study, we hope that future efforts will follow and provide detailed insight into observed changes in proteasome biology as a foundation for future understanding.

## Experimental procedures

### Mice

All animal studies were performed with protocols that are approved by the Institutional Animal Care and Use Committees of The Johns Hopkins University School of Medicine. Wild-type C57BL/6 mice were purchased from Charles River Laboratories (Stock number 027; RRID: IMSR_CRL:27). The 21-day-old male mice were used for brain and liver tissue collection. Embryos from the 17.5 days of gestation pregnant wild-type C57BL/6 animals were used for preparing primary neuronal cultures. Mice were euthanized with CO_2_-induced anoxia and decapitated.

### Rhesus macaque

All procedures were performed in accordance with the *NIH’s Guide for the Care and Use for Laboratory Animals*, the *US Department of Agriculture Animal Welfare Act*, and under the approval of the Johns Hopkins Medical School Animal Care and Use Committee. The rhesus macaque used for this study was a 14-month-old female (125J) fed a balanced macaque diet (Purina Animal Nutrition, Gray Summit, MO) and housed with related conspecifics with indoor/outdoor access in compliance with federal animal welfare regulations. This macaque was originally meant for a simian immunodeficiency virus (SIV) study and remained uninfected for SIV. Criteria for humane euthanasia prior to planned endpoint if any of the following were observed: (a) weight loss of greater than 15%, (b) CD4+ T-lymphocytes count less than 5% baseline level, (c) clinical signs of neurological disease, (d) intractable diarrhea, and opportunistic infection.

The rhesus macaque used in this study (125J) was humanely euthanized by the Retrovirus Laboratory of the Department of Molecular and Comparative Pathobiology at Johns Hopkins University School of Medicine at 14 months old due to chronic diarrhea and a thin body condition score after unsuccessful steroid treatment. Necropsy protocols were performed in accordance with previous work routinely performed in accordance with the American Veterinary Medical Association guidelines for the euthanasia of animals (157, 158, 159, 160). Euthanasia occurred with an overdose of sodium pentobarbital while under ketamine sedation (15- to 20- mg/kg intramuscular injection) before perfusion with phosphate-buffered saline (PBS) (Gibco) to remove blood from tissues as described. After harvest, the whole brain was placed in cold 2.5% agarose for ease of slicing, and 4-mm coronal sections were cut, microdissected by brain region, and flash frozen in liquid nitrogen.

### Neuronal cultures

For primary mouse neuronal cortical cultures, pregnant wild-type C57BL/6 mice were euthanized and decapitated after 17.5 days of gestation, and neurons were plated from dissected embryos of mixed sex as described previously (161). Briefly, brains were removed from isolated embryos, and dissected cortices were then moved into dissociation buffer (10 mM MgCl_2_, 10 mM HEPES,1 mM kynurenic acid in Hank’s Balanced Salt Solution) supplemented with 16.67 U/ml papain (Worthington; LS003127), which was incubated for 45 min at 37 °C. Proteolyzed tissue was then rinsed in 10 mg/ml trypsin inhibitor (Millipore; T9253) twice for 5 min and then resuspended and mechanically dissociated in Neurobasal media (Thermo Fisher; 21103049) into a single cell suspension. Cultured cortical neurons were counted by hemocytometer, and 1.5 million cells were plated on 6-well plates pre-coated with 1 mg/ml poly-L-lysine dissolved in 1 M Tris pH 8.0. Cultures were maintained in Neurobasal media supplemented with 2% B-27 (ThermoFisher; 17504044), penicillin/streptomycin (100 U/ml and 100 μg/ml, respectively) (Millipore; 15140122), and 2 mM glutamine at 37 °C/5% CO_2_. Culture media were changed to Neurobasal media without serum 3 days after plating and supplemented with fresh media every 4 to 5 days. All experiments were performed at days *in vitro* (DIV) 12.

### Human brain tissue

The post-mortem brain samples were supplied by the Lieber Institute for Brain Development Human Brain Repository, all collected with an informed consent of the next of kin. Cases for this study were collected from the Office of the Chief Medical Examiner for the State of Maryland under the State of Maryland Department of Health and Mental Hygiene Protocol 12-24, and from the Western Michigan University Homer Stryker M.D. School of Medicine Department of Pathology, Department of Pathology University of North Dakota School of Medicine and Health Sciences, and Santa Clara County California Medical Examiners’ Office under Western Institutional Review Board Protocol 20111080. All samples were collected and processed using a rigorous standardized protocol specifically developed to minimize sample heterogeneity and technical artifacts. In the current study, we used only samples from Caucasian individuals to further minimize sample heterogeneity and maximize the power of downstream analyses. The procedures for sample collection, curation, and diagnosis have been described in detail elsewhere (162). Briefly, clinical and demographic information was gathered by the review of medical and psychiatric records (163) and *via* a structured interview (164, 165, 166) with next of kin conducted within 1 week of donation. Psychiatric narratives were prepared for each case, summarizing information obtained from all available sources, and each case was independently reviewed by two board-certified psychiatrists who arrived at consensus DSM-IV Axis I lifetime diagnoses (167). History of cigarette smoking at the time of death was collected during the initial telephone screening and verified through toxicological analysis of nicotine and cotinine levels in the blood or brain tissue. Toxicological analyses of blood, vitreous humor fluid, occipital pole, and/or urine were conducted by a forensic toxicologist. A neuropathological examination was performed on each case by a board-certified neuropathologist. Subjects with evidence of clinically or neuropathologically significant cerebrovascular disease (infarcts or hemorrhages), subdural hematoma, primary parenchymal brain tumors, or other significant pathological features were excluded from further study. Subjects with acute subarachnoid hemorrhages that were directly related to the immediate cause of death were not excluded. The cause and manner of death and any contributory causes were obtained from medical examiner documents. Neurotypical controls had no history of significant psychiatric symptoms or substance abuse, as determined by both telephone interviews with next of kin and medical examiner documentation. By definition, cases were excluded from the neurotypical control group if the manner of death was suicide, if death was due to drug overdose or poisoning, or if toxicology results were positive. The agonal state was assessed by gathering data regarding specific medical conditions and treatment proximate to the date of death (*e.g.*, coma, hypoxia, seizures) and the duration of the terminal phase. Age at death was verified by obtaining both date of birth and date of death through medical records, medical examiner documents, and family interviews. Post-mortem interval was defined as the time elapsed, in hours, between the pronounced time of death and time of tissue freezing.

The cortical ribbon from the parahippocampal gyrus was dissected under visual guidance using a hand-held dental drill at the level of the pulvinar and lateral geniculate body. To avoid elements of the subicular complex, the dissection was directed toward the collateral sulcus. The primary visual cortex (BA17) was identified on the medial aspect of the rostral face of the slab containing the occipital pole. Features used to identify BA17 included the calcarine sulcus and the line of Gennari, which are easily seen on visual inspection. The full thickness of the cortical ribbon was dissected.

All post-mortem brains of subjects (controls, preclinical AD, and AD) have been sampled for neuropathology and specific Alzheimer’s lesions: beta-amyloid plaques, neurofibrillary tangles, and tau-positive neurites. Samples were also genotyped for the apolipoprotein E gene. The sampled tissue sections were fixed in 10% buffered formalin and paraffin-embedded into blocks for microscopic analysis (10-micron thickness). Sections included the superior frontal gyrus, middle and superior temporal gyri, inferior parietal cortex, occipital cortex, hippocampal formation and entorhinal cortex, midbrain, pons, medulla, and cerebellum (including cerebellar cortex and deep cerebellar nuclei). Sections were silver-stained using the Hirano method (168) and immune stained using antibodies against ubiquitin, phosphorylated anti-tau (PHF-1), and beta-amyloid protein (6E10). Microscopic preparations were examined using conventional light microscopy. Alzheimer’s neuropathology ratings include the Braak (169) staging schema evaluating tau neurofibrillary tangle burden and the CERAD scoring system as a measure of senile plaque burden (neuritic and diffuse). An Alzheimer’s likelihood diagnosis was then performed based on the published consensus recommendations for post-mortem diagnosis of Alzheimer’s disease (170) in prior publications (169, 170, 171, 172). Preclinical AD includes subjects with positive AD neuropathology (Braak and CERAD score) over the age of 50, with no signs of cognitive decline associated with AD.

### Antibodies

The following antibodies were used according to the manufacturer’s suggestions for immunoblotting: anti-α1–7 proteasome subunit (Enzo; BML-PW8195-0025), anti-β5 proteasome subunit (Enzo; BML-PW8895-0010), anti-β2 proteasome subunit (Enzo; BML-PW9300-0025), anti-β-actin (Abcam; ab8226), anti-NR2B (Thermo Fisher Scientific; MA1-2014), anti-Ubiquitinylated proteins, clone FK2 (Millipore Sigma; 04-263), anti-Rpt5/S6a 19S proteasome subunit (Enzo; BML-PW8310-0025), anti-Tau-5 (Thermo Fisher Scientific; AHB0042), anti-rabbit IgG HRP-linked antibody (Cell Signaling; 7074), anti-mouse IgG HRP-linked antibody (Cell Signaling; 7076).

### Sample preparation for in-gel detection of labeled active proteasome subunits of purified proteasome, mouse primary cortical cultures, mouse brain/liver tissue, and human brain tissue

For *in vitro* experiments performed with purified human 20S (Enzo; BML-PW8720) or 26S proteasome (Enzo; BML-PW9310), 0.25 μg proteasome was incubated with vehicle (DMSO) or 0.5 μM MV151 (or MV152, as a negative control; gifts from Dr Alexei Kisselev, Auburn University) in the presence or absence of 0.5 μM epoxomicin (Millipore; E3652) (or MG132 (Cell Signaling; 2194), or 0.1 μM LU-001c (gift from Dr Alexei Kisselev) where indicated) for 1 h at 37 °C. All vehicle treatments were performed with 1% final DMSO in the reaction mixture, and all inhibitors were added into the reaction mixture 15 min before the MV151 addition. Samples were split into two, and half of the reaction mixture was treated with Laemmli SDS sample buffer for denaturing SDS-PAGE gel experiments. The rest of the sample was treated with native loading dye (250 mM Tris pH 7.4, 50% (v/v) glycerol, 0.007% xylene cyanol) to assess the activity and abundance of distinct proteasome compositions *via* native-PAGE experiments, as described below. Reaction mixtures treated with Laemmli SDS sample buffer were boiled at 95 °C for 5 min and loaded on gels. For in cell MV151 concentration titration experiments, DIV12 mouse primary cortical cultures were treated with the indicated concentration of MV151 for 1 h at 37 °C. For the time course experiments, neurons were treated with 1 μM MV151 for indicated time points, and samples were collected at corresponding time points, followed by immediate quenching of the reaction with sample buffer. For the Suc-LLVY-AMC and MV151 comparison experiments, neurons were incubated with vehicle (1% DMSO) or 1 μM MV151 in the presence or absence of 2.5 μM epoxomicin for 1 h. Epoxomicin treatment was performed 15 min prior to MV151 incubation. At the end of the treatment, neurons were rinsed once with 1× PBS (Thermo Fisher; 10010049) and collected in 250 μl MV151 lysis buffer (50 mM tris pH 7.5, 250 mM sucrose, 5 mM MgCl_2_, 2 mM ATP, 1 mM DTT). Upon collection, samples were split into two, treated with either sample buffer or native loading dye, and loaded on corresponding gels. Brain and liver tissue were dissected from P21 male mice for mouse tissue experiments. 30 mg whole cortical or liver tissue was carefully homogenized in 0.3 ml MV151 lysis buffer with Dounce tissue homogenizer. Homogenized brain tissue was spun down at 50,000 r.p.m. for 45 min at 4 °C. Protein concentration was quantified by the Bradford assay (Bio-Rad; 5000201). For the *in vitro* labeling reactions of the tissue lysate, 20 μg total protein lysate was incubated with vehicle (1% DMSO) or 0.5 μM MV151 in the presence or absence of 0.25 μM epoxomicin for 1 h at 37 °C in a total reaction volume of 20 μl. Half of the reaction mixture was treated with sample buffer, and the other half was treated with native loading dye. For the tissue lysate MV151 time course experiments, the reaction of 20 μg total protein lysate treated with 0.5 μM MV151 was stopped at indicated time points by the addition of sample buffer and loaded on SDS-PAGE gels. Each tissue type was normalized to its own actin levels, which served as an internal control for sample preparation, gel loading, and other potential experimental anomalies. Generally, for the given amount of protein loaded (20 μg) for each sample prepared we observed very similar actin levels. Comparison between liver and cortical tissue subunit activity was based on the actin-normalized MV151 signal at indicated time points. Finally, 30 mg human brain tissue (parahippocampal gyrus; PHG and visual cortex; VC) (from The Lieber Institute for Brain Development brain repository) was processed the same way as the mice tissue samples.

### In-gel activity assays and immunoblot analysis

For in-gel activity assay and immunoblotting of 12% denaturing SDS-PAGE, Tris/Glycine gels were made in the laboratory, and SDS-treated samples were run at 110 V for 120 min. In-gel visualization of the MV151 signal was performed with wet gel slabs using Cy3/Tamra setting (λ_ex_: 532, λ_em_: 560) on the Typhoon Variable Mode Imager. The MV151 signal intensity from individual three bands (β2, β1, β5) was measured in ImageJ to obtain the saturation curves in time course experiments. Gels were then transferred to nitrocellulose membranes at 60 mA overnight in 20% methanol-containing transfer buffer. All antibodies were made up in 5% BSA in 0.1% 1× TBS plus 0.1% Tween-20 (TBST). Membranes were incubated with primary antibodies raised against indicated proteins overnight at 4 °C. Membranes were washed five times for 5 min with TBST, then incubated with secondary antibodies conjugated to horseradish peroxidase (HRP) for 1 h at room temperature with gentle shaking, washed 5 times for 5 min with TBST, and incubated with ECL. Images were exposed on film and were quantified with ImageJ by standard densitometry analysis where indicated. Actin was used as a loading control.

### Native gel and Suc-LLVY-AMC proteasome activity assay

For in-gel activity assay and immunoblotting native-PAGE gels, 4% non-denaturing gels were made in the laboratory and run at 130 V for 180 min using native gel running buffer (0.15 M tris base, 0.15 M boric acid, 1 mM EDTA, 2.5 mM MgCl_2_, supplemented with 0.5 mM ATP and 1 mM DTT). In-gel visualization of the MV151 signal in native gels was performed with wet gel slabs using Cy3/Tamra setting (λ_ex_: 532, λ_em_: 560) on the Typhoon Variable Mode Imager. In-gel activity was then detected by hydrolysis of 100 μM proteasome fluorogenic substrate (Suc-Leu-Leu-Val-Tyr-AMC, Bachem; I-1395.0100). Gels were incubated with Buffer A (25 mM Tris pH 7.4, 10 mM MgCl_2_, 10% glycerol, 1 mM ATP, 1 mM DTT) supplemented with 100 μM Suc-LLVY-AMC in the presence or absence of 0.02% SDS in the dark for 30 min at 30 °C as indicated in figure legends. Gels were imaged under ultraviolet light with the Gel Doc AZ system to detect the AMC signal. Next, gels were transferred to PVDF membranes, pre-activated with methanol, at 60 mA overnight in a transfer buffer. The rest of the immunoblot protocol for the native gel is the same as described above.

### *In vitro* proteasome activity assay

The AMC conjugated peptide, Suc-LLVY-AMC (Enzo; BML-P802-9090) was used to assess the chymotrypsin-like proteasome activity. The purified human proteasome (0.1 μM) or tissue homogenate (5 μg per reaction) was diluted in assay buffer (50 mM HEPES, 20 mM KCl, 5 mM MgCl_2_, 1 mM DTT, 10 mM ATP (pH 7.4), 0.03% SDS). To control for the non-proteasomal-mediated cleavage of substrates, 0.5 μM epoxomicin, MG132, MV151, or MV152 was added to the reaction mixture for 10 min, followed by the addition of 75 μM Suc-LLVY-AMC substrate. The activity was monitored by continuous fluorescent measurement of the released AMC at 30 °C at 1.5 to 2 min intervals for 10 min or 1 h using a microplate fluorescence reader (Infinite 200 PRO; λ_ex_: 360, λ_em_: 460).

### Quantification and statistical analysis

All statistical analysis was performed using GraphPad Prism Software (Version 9.3.0 (345), GraphPad). Statistical tests were applied to all experiments where applicable. The statistical tests performed for each experiment are indicated in the respective figure legends. Two-way ANOVA was used to determine the difference between MV151 incorporation dynamics to distinct active subunits across different tissue types. Multiple variable correlation analysis (two-tailed; 95% CI) with Pearson correlation coefficient (r) was used to determine the correlation between 20S activity (MV151 signal), Tau protein abundance (normalized to actin), and Braak stage in human brain tissue samples. One-way ANOVA was used to determine the significance between densitometric quantification of indicated proteins/activities in the control, preclinical AD, and AD human brain tissue. Mann–Whitney *U* test (native sample analysis) and Student’s unpaired *t* test (denatured sample analysis) were used to determine the significance between healthy controls and cases with severe AD. Significance was indicated in the figures as adjusted *p*-values for two-way ANOVA ∗*p* < 0.0332, ∗∗*p* < 0.0021, ∗∗∗*p* < 0.0002, ∗∗∗∗*p* < 0.0001 and as ∗*p* < 0.05, ∗∗*p* < 0.02 for the rest of the tests. Data that are not labeled with asterisks did not reach statistical significance.

## Data and software availability

All reagents and protocols used in this study are available for sharing upon reasonable request to the authors.

## Supporting information

This article contains supporting information.

## Inclusion and diversity

The authors routinely considered diversity and inclusivity.

## Conflict of interest

The authors declare that they have no conflicts of interest with the contents of this article.

## Figures and Tables

**Supplemental Figure S7:**