# Supplementary material for: Orthogonal approaches required to measure proteasome composition and activity in mammalian brain tissue
Source: J Biol Chem. 2023 May 11;299(6):104811. doi: 10.1016/j.jbc.2023.104811 (PMC10276296; doi:10.1016/j.jbc.2023.104811)
Supplement: Supplemental Figures and Table Legend [file mmc1.docx]

**SUPPLEMENTAL INFORMATION**

**Table S1**

**Figures S1 – S7**

**Table S1. Age, Braak stage, CERAD rating B/C rating, post-mortem interval, race, sex, smoking habit, brain region, APOE genotype, RIN value for all human samples (related to Figures 6 and 7).**

AD, Alzheimer’s disease; PRE-AD: Pre-clinical Alzheimer’s disease; Braak stage, B3: V-VI, B2: III-IV, B1: I-II, B0: no Tau tangle detection; CERAD ratings: C0-none, C1-sparse, C2-moderate, C3-frequent; AP, Alzheimer’s pathology; Interm, intermediate; Prob, probable; Def, definite; Poss: possible; PMI: post-mortem interval; CAUC, Caucasian; M, male; PHG, parahippocampal gyrus; VC, visual cortex; APOE: Apolipoprotein E; RIN: RNA Integrity Number

**Figure S1. MV151 is a robust tool for detecting β-subunit activity in purified proteasomes, related to Figure 1.**

**(A)** Purified human 20S or 26S proteasome was incubated with vehicle (DMSO), MV151, or an inactive form of MV151 (MV152). MV151-treated samples were incubated with vehicle (DMSO) or proteasome inhibitor MG132. Top: Samples were applied to denaturing SDS-PAGE and imaged with the Cy3 channel to detect MV151 labeling. Note that 26S lanes are shown cropped in Figure 1D. Bottom: MV151 gels were immunoblotted with antibodies to the indicated core proteasome subunits (β2, β5), a 19S regulatory particle (Rpt5/S6) subunit, and actin. ** denotes MV151-bound active subunits, 1.1 kDa-shifted band.

**(B)** Purified human 20S proteasome was incubated with vehicle (DMSO), epoxomicin (Epox), MV151, or MG132. Top: Samples were applied to denaturing SDS-PAGE and imaged with the Cy3 channel to detect MV151 labeling. Bottom: MV151 gels were immunoblotted with antibodies raised against β5 and Rpt5/S6. ** denotes MV151-bound active subunits, 1.1 kDa-shifted band; * denotes epoxomicin-bound active subunit, 0.5 kDa-shifted band.

**(C)** Purified human 20S proteasome was incubated with vehicle (DMSO) or MV151 in the presence or absence of 0.1 µM LU-001c β1 subunit-specific inhibitor for 1 hr. Samples were applied to denaturing SDS-PAGE and imaged with the Cy3 channel to detect MV151 labeling.

**Figure S2. Kinetics of proteasome catalytic β subunit activity** **are measurable using MV151 in mouse primary cortical cultures, related to Figure 2.**

**(A)** DIV12 mouse primary cortical neurons were treated with indicated MV151 concentrations for 1 hr. Cells were collected and lysed for SDS-PAGE. Gels were imaged with the Cy3 channel for the detection of MV151 incorporation levels. MV151 bands correspond to individual catalytically active β subunits (arrows).

**(B)** DIV12 mouse primary cortical neurons were incubated with MV151. Samples were collected at indicated times and separated by SDS-PAGE. Gels were imaged with the Cy3 channel for the detection of MV151 incorporation levels. MV151 bands correspond to individual catalytically active β subunits (arrows).

**(C)** Top: DIV12 mouse primary cortical neurons were incubated with MV151. Samples were collected at indicated times and separated by SDS-PAGE. Gels were imaged with the Cy3 channel for the detection of MV151 incorporation levels. MV151 bands correspond to individual catalytically active β subunits (arrows). Bottom: MV151 gels were immunoblotted with antibodies to the indicated core proteasome subunits (β2, β5) and actin. ** denotes MV151-bound active subunits, 1.1 kDa-shifted band.

**(D)** Quantification of actin-normalized MV151 signals from (C). Data points are represented as mean ± SEM (n = 3).

**(E)** Quantification of % active β2 (top) and β5 (bottom) subunits was performed by calculating the ratio of MV151-bound β2 (or β5) band intensity (**) to total β2 (or β5) band intensity in corresponding immunoblots. Data are represented as mean ± SEM.

**Figure S3. MV151 is an efficient probe for measuring the activity of doubly-capped (30S), singly-capped (26S), and 19S-uncapped (20S) proteasome complexes from intact live neurons to a similar extent as with tissue homogenates, related to Figure 3.**

**(A)** Purified human 20S or 26S proteasomes were incubated with vehicle (DMSO) or MV151. (1) To determine the relative activity of distinct proteasome complexes, samples were separated using 4% native-PAGE and imaged with the Cy3 channel to detect the MV151 incorporation level (yellow). (2) The gels were then imaged under UV light before incubating the gel with Suc-LLVY-AMC substrate to show the bleed-through of the MV151 Cy3 signal under UV light (yellow). (3) Gels were then treated with Suc-LLVY-AMC (AMC) and imaged under UV light to monitor the proteasome chymotrypsin-like activity (purple). Cy3 and UV bands correspond to individual catalytically active proteasome complexes (30S, 26S, 20S) (arrows). Note the reduced AMC activity with purified 20S compared to 26S. Note the reuse of the same figure from Figure 3A for this panel's in-gel fluorescence detection images (left) to show the Cy3 bleed-through of the gel before and after Suc-LLVY-AMC incubation. Below: Native gels were immunoblotted with antibodies to the indicated core proteasome subunits (α1-7**,** β5). Cy3, UV, and immunoblot bands correspond to individual proteasome complexes (30S, 26S, 20S) (arrows). Note the presence of PA28 activator that is present in the purified 20S proteasome complexes.

**(B)** Proteasome activity in purified human 20S proteasome in the presence or absence of 0.5 µM MV152, MV151, epoxomicin (Epox), MG132 was measured by the cleavage of Suc-LLVY-AMC in the presence (top) or absence (bottom) of 0.03% SDS for 1 hr using a microplate fluorescence reader. Slopes were calculated by using the time points in which the reaction is linear to calculate the rate of proteolysis. Data are represented as mean ± SEM.

**(C)** DIV12 cortical cultures were incubated with vehicle (DMSO) or MV151 in the presence or absence of epoxomicin (Epox). To determine the relative activity of distinct proteasome complexes, samples were separated using 4% native-PAGE. First, native gels were imaged with the Cy3 channel to detect MV151 incorporation (yellow). Second, native gels were treated with Suc-LLVY-AMC (AMC) and imaged under UV light to monitor the proteasome chymotrypsin-like activity (purple). Imaged gels were then immunoblotted with antibodies to the indicated core proteasome subunits (α1-7), a 19S regulatory particle subunit (Rpt5/S6), or ubiquitin. Cy3, UV, and immunoblot bands correspond to individual proteasome complexes (30S, 26S, 20S) (arrows). Note that there is a low amount of bleed-through in the AMC assay. This is due to the low MV151 signal.

**(D)** Samples from (**C**) were applied to denaturing SDS-PAGE and imaged with the Cy3 channel to detect MV151 labeling. MV151 gels were then immunoblotted with antibodies to the indicated core proteasome subunits (α1-7, β2, β5), a 19S regulatory particle subunit (Rpt5/S6), actin, or ubiquitin. * denotes epoxomicin-bound active subunit, 0.5 kDa-shifted band; ** denotes MV151-bound active subunits, 1.1 kDa-shifted band. Note the increase in ubiquitin signal with the treatment of epoxomicin or MV151.

**Figure S4. Human brain tissue contains only detectable 19S-uncapped 20S proteasome regardless of age or sex of subjects.**

(**A-B**) Frozen human brain samples with standard PMI values were collected and applied to native (**A or B, top**) or denaturing (**A or B, bottom**) gel conditions. For native gels samples were prepared for AMC assays as previously described, followed by immunoblot analysis using indicated antibodies. For denaturing gels, samples were prepared in denaturing buffer as previously described, followed by SDS-PAGE and immunoblot analysis using indicated antibodies. Detectable proteasome complex signal appears at locations indicated for the different 19S-uncapped (20S), singly capped (26S), or doubly capped (30S) proteasome.

**Figure S5. Schematic diagram of the workflow for assessing the activity, abundance, and composition of the proteasome in brain samples (parahippocampal gyrus (PHG) and visual cortex (VC)) from unaffected healthy subjects or patients that have been diagnosed with Alzheimer’s disease (AD), related to Figure 5, 6 and 7.**

(**A)** Frozen human brain tissue from 30 PHG and 30 VC samples were lysed in MV151 lysis buffer and gently homogenized with Dounce tissue homogenizer. Tissue homogenate was then incubated with MV151 for 1 hr at 37 °C. Finally, samples were split and prepared for our two orthogonal approaches: 1) Native conditions for analysis of activity, abundance, and composition of human proteasome complexes (30S/26S/20S); 2) Denaturing conditions for analysis of catalytic β subunit (β1, β2, β5) activity and kinetics. Created with BioRender.com.

**(B)** Summary of patient demographics shown in Supplementary Table 1.

**Figure S6. Pearson correlation coefficient for the relation of proteasome activity (MV151), abundance (β5), Tau levels, and Braak stage, related to Figure 6.**

**(A)** Parahippocampal gyrus human brain samples were immunoblotted with Tau and actin. Representative immunoblots are shown.

**(B)** Left: Pearson r heatmaps with indicated correlation coefficients (r) showing the relation of Tau levels, normalized 20S MV151 signal from Figure 5, and Braak stage in PHG. Significant correlations are denoted with asterisks (*: p < 0.05, **: p < 0.02). Right: Pearson r heatmaps with indicated correlation coefficients (r) showing the relation of Tau levels, 20S β5 band intensity from Figure 5, and Braak stage in PHG. Significant correlations are denoted with asterisks (*: p < 0.05, **: p < 0.02).

**(C)** Visual cortex human brain samples were immunoblotted with Tau and actin. Representative immunoblots are shown.

**(D)** Left: Pearson r heatmaps with indicated correlation coefficients (r) showing the relation of Tau levels, normalized 20S MV151 signal from Figure 5, and Braak stage in VC. Right: Pearson r heatmaps with indicated correlation coefficients (r) showing the relation of Tau levels, 20S β5 band intensity from Figure 5, and Braak stage in VC.

**Figure S7. Kinetics of catalytic proteasome β subunits in VC brain tissue from healthy control cases compared to AD patients, related to Figure 7.**

**(A**) Top: VC human brain tissue homogenate from healthy control cases was treated with MV151, and samples were collected at indicated time points. Samples were applied to SDS-PAGE and imaged with the Cy3 channel to detect MV151 labeling. Bottom: MV151 gels were immunoblotted with antibodies to the indicated core proteasome subunits (β2, β5), actin, or Tau. β2 and β5 blots show unmodified subunit (arrow), ~0.5 kDa epoxomicin-shifted subunit (*), and ~1.1 kDa MV151-shifted subunit (**). MV151 bands correspond to individual catalytically active β subunits (arrows). Note: we detect inducible β2 (β2i) and β5 (β5i) subunits.

**(B)** Top: Cy3 in-gel visualization of MV151 time course with VC human brain tissue homogenate from AD-diagnosed cases treated as in (A). Bottom: MV151 gels were immunoblotted with antibodies to the indicated core proteasome subunits (β2, β5), actin, or Tau. β2 and β5 blots show unmodified subunit (arrow) and ~1.1 kDa MV151-shifted subunit (**). MV151 bands correspond to individual catalytically active β subunits (arrows). Note: we detect inducible β2 (β2i) and β5 (β5i) subunits.

**(C)** Densitometric analysis of MV151 Cy3 signal for individual β subunits. Data are represented as mean ± SEM. Statistical test: Student’s unpaired t-test (*: p < 0.05).

**(D-E)** Analysis of β5 and β2 protein levels in control and AD cases via densitometric analysis of immunoblots. Actin was used as a loading control. Data are represented as mean ± SEM. Statistical test: Student’s unpaired t-test.

**(F-G)** Quantification of % active β5 (F) and β2 (G) subunits was performed by calculating the ratio of MV151-bound β5 (or β2) band intensity (**) to total β5 (or β2) band intensity in corresponding immunoblots. Data are represented as mean ± SEM. Statistical test: Student’s unpaired t-test (*: p < 0.05).
