# Supplementary material for: Orthogonal approaches required to measure proteasome composition and activity in mammalian brain tissue
Source: J Biol Chem. 2023 May 11;299(6):104811. doi: 10.1016/j.jbc.2023.104811 (PMC10276296; doi:10.1016/j.jbc.2023.104811)
Supplement: Supplemental Table S1 [file mmc2.docx]

**Supplementary Table 1**

|  | Age | Braak | CERAD | B/C Rating | PMI | Race | Sex | Smoking | Region | APOE | RIN |
| --- | --- | --- | --- | --- | --- | --- | --- | --- | --- | --- | --- |
| AD | 80.84 | B3 | C2 | Interm AP | 23.00 | CAUC | M | No | PHG, VC | E4, E4 | 6.00 |
| AD | 90.47 | B3 | C3 | Prob AD | 35.00 | CAUC | M | No | PHG, VC | E3, E3 | 7.10 |
| AD | 76.33 | B2 | C2 | Prob AD | 28.50 | CAUC | M | No | PHG, VC | E3, E3 | 7.30 |
| AD | 89.38 | B3 | C1 | Low AP | 29.50 | CAUC | M | No | PHG, VC | E3, E3 | 6.50 |
| AD | 96.01 | B3 | C2 | Interm AP | 30.50 | CAUC | M | No | PHG, VC | E3, E3 | 6.30 |
| AD | 87.41 | B3 | C3 | Def AD | 48.00 | CAUC | M | No | PHG, VC | E3, E3 | 7.30 |
| AD | 89.28 | B3 | C2 | Prob AD | 32.50 | CAUC | M | No | PHG, VC | E3, E4 | 6.70 |
| AD | 79.40 | B3 | C3 | Def AD | 44.00 | CAUC | M | No | PHG, VC | E3, E4 | 6.40 |
| AD | 77.15 | B3 | C3 | Def AD | 24.50 | CAUC | M | Yes | PHG, VC | E4, E4 | 6.40 |
| AD | 79.79 | B3 | C2 | Interm AP | 51.00 | CAUC | M | Yes | PHG, VC | E3, E4 | 6.83 |
| PRE-AD | 71.86 | B2 | C2 | Interm AP | 36.00 | CAUC | M | No | PHG, VC | E3, E4 | 7.60 |
| PRE-AD | 71.20 | B2 | C2 | Interm AP | 33.50 | CAUC | M | Yes | PHG, VC | E2, E3 | 7.50 |
| PRE-AD | 62.58 | B3 | C2 | Interm AP | 33.50 | CAUC | M | Yes | PHG, VC | E3, E4 | 6.80 |
| PRE-AD | 67.34 | B2 | C2 | Interm AP | 31.00 | CAUC | M | No | PHG, VC | E3, E4 | 6.00 |
| PRE-AD | 65.28 | B3 | C1 | Low AP | 30.50 | CAUC | M | No | PHG, VC | E3, E3 | 7.60 |
| PRE-AD | 64.88 | B3 | C2 | Poss AD | 33.50 | CAUC | M | No | PHG, VC | E3, E4 | 7.40 |
| PRE-AD | 87.22 | B1 | C2 | Prob AD | 23.50 | CAUC | M | No | PHG, VC | E3, E3 | 7.20 |
| PRE-AD | 70.00 | B2 | C2 | Interm AP | 24.00 | CAUC | M | Yes | PHG, VC | E3, E4 | 8.00 |
| PRE-AD | 76.30 | B2 | C2 | Interm AP | 39.50 | CAUC | M | Yes | PHG, VC | E3, E3 | 6.70 |
| PRE-AD | 62.37 | B3 | C2 | Low AP | 21.00 | CAUC | M | Yes | PHG, VC | E4, E4 | 6.20 |
| CONTROL | 63.16 | B2 | C0 | No AP | 41.00 | CAUC | M | No | PHG, VC | E3, E3 | 6.00 |
| CONTROL | 72.43 | B0 | C0 | No AP | 27.50 | CAUC | M | Yes | PHG, VC | E1, E3 | 7.40 |
| CONTROL | 86.87 | B0 | C0 | No AP | 27.00 | CAUC | M | No | PHG, VC | E3, E3 | 7.00 |
| CONTROL | 82.26 | B2 | C0 | No AP | 22.50 | CAUC | M | No | PHG, VC | E3, E3 | 6.40 |
| CONTROL | 84.52 | B2 | C0 | No AP | 22.00 | CAUC | M | No | PHG, VC | E2, E3 | 6.20 |
| CONTROL | 67.75 | B2 | C0 | No AP | 30.50 | CAUC | M | No | PHG, VC | E2, E2 | 6.80 |
| CONTROL | 81.65 | B2 | C1 | Low AP | 21.00 | CAUC | M | No | PHG, VC | E3, E4 | 7.30 |
| CONTROL | 65.59 | B0 | C0 | No AP | 27.00 | CAUC | M | Yes | PHG, VC | E3, E4 | 6.00 |
| CONTROL | 70.88 | B1 | C1 | Low AP | 22.00 | CAUC | M | No | PHG, VC | E3, E3 | 6.40 |
| CONTROL | 75.97 | B2 | C1 | Low AP | 32.50 | CAUC | M | Yes | PHG, VC | E3, E3 | 6.00 |
